# Comprehensive Analyses of NAC Transcription Factor Family in Almond (*Prunus dulcis*) and Their Differential Gene Expression during Fruit Development

**DOI:** 10.3390/plants10102200

**Published:** 2021-10-16

**Authors:** Zeeshan Zafar, Sidra Fatima, Muhammad Faraz Bhatti

**Affiliations:** Atta-ur-Rahman School of Applied Bioscience, National University of Sciences and Technology, H-12, Kashmir Highway, Islamabad 44000, Pakistan; zeeshanzafar98@hotmail.com (Z.Z.); sidrafatima49032@yahoo.com (S.F.)

**Keywords:** genome wide identification, NAC, transcription factor, phylogenetic analysis, syntenic analysis, gene duplication, RNA-seq, differential gene expression

## Abstract

As plant specific transcription factors, NAC (NAM, ATAF1/2, CUC2) domain is involved in the plant development and stress responses. Due to the vitality of NAC gene family, BLASTp was performed to identify NAC genes in almond (*Prunus dulcis*). Further, phylogenetic and syntenic analyses were performed to determine the homology and evolutionary relationship. Gene duplication, gene structure, motif, subcellular localization, and *cis*-regulatory analyses were performed to assess the function of *PdNAC*. Whereas RNA-seq analysis was performed to determine the differential expression of *PdNAC* in fruits at various developmental stages. We identified 106 NAC genes in *P. dulcis* genome and were renamed according to their chromosomal distribution. Phylogenetic analysis in both *P. dulcis* and *Arabidopsis thaliana* revealed the presence of 14 subfamilies. Motif and gene structure followed a pattern according to the *PdNAC* position in phylogenetic subfamilies. Majority of NAC are localized in the nucleus and have ABA-responsive elements in the upstream region of *PdNAC*. Differential gene expression analyses revealed one and six *PdNAC* that were up and down-regulated, respectively, at all development stages. This study provides insights into the structure and function of *PdNAC* along with their role in the fruit development to enhance an understanding of NAC in *P. dulcis*.

## 1. Introduction

Transcription factors have immense importance due to their role in controlling the transcription rate by binding to the *cis*-regulatory promoter elements [1]. DNA binding domain of transcription factors determines their function in gene expression regulatory networks. Transcription factors bind to the *cis*-regulatory elements resulting in the regulation of the targeted gene for enhance or reduced expression level. In plants, transcription factors are divided into several categories based on the DNA binding domain. These families are *bZIP*, *NAC*, *MYB*, *DREB*, *WRKY*, *AP2*/*EREBP*, C_2_H_2_, and others [2]. Plant’s growth and development depend on the action of these transcription factors in various ways such as hormone signaling, secondary metabolism, organ formation and response to the environment [3,4]. Similarly, numerous plant transcription factors are responsible for the improvement of plant tolerance against the abiotic stresses [5].

NAC are major plant transcription factors having more than 100 genes in rice and *Arabidopsis thaliana* [6,7]. NAC family name is based on three proteins: no apical meristem (NAM), ATAF, and cup-shaped cotyledon (CUC), all contain a similar DNA binding domain. NAC TFs were originally identified as *Petunia* NAM, and *Arabidopsis* CUC [8,9]. NAC contains a conserved NAC domain consisting of highly conserved N-terminal that functions as DNA binding domain involved in oligomerization of NAC into dimers [5,10]. Whereas NAC contains a more diverse and intrinsically disorder C-terminal functioning as transcription regulatory domain [10,11]. NAC TFs are divided into typical and atypical classes where typical NAC contains conserved NAC domain at N-terminal and a divergent C-terminal region. Whereas atypical NAC contains a conserved C-terminal region or no C-terminal at all in addition to the conserved NAC domain at N-terminal [5].

NAC transcription factors have a major role in growth and environmental adaptation of plants along with abiotic stress responses [5,12,13,14]. It has been found that many NAC TFs play a vital role in plant immunity [15,16,17]. In susceptible and resistant crops, it has been found that six NAC TFs are involved in response to tomato yellow leaf curl virus (TYLCV) infecting tomato plants [18]. Secondary cell wall biosynthesis is also regulated by NAC TFs [19,20,21]. NAC TFs have been found to enhance tolerance to cold stress, drought, and salt stress as overexpression of *OsNAC6* in rice [22,23,24]. Overexpression of JUB1 NAC TF increases salinity tolerance in tomato [25]. Furthermore, a membrane associated NAC TFs was involved in thermotolerance in rice [26]. In grapevine, NAC TFs were responsible for the leaf senescence [27]. It has been found that *ANAC096* (*A. thaliana*) cooperates with the bZIP-type TF ABRE binding factor and binding domain (ABF/AREB) for plant’s survival under osmotic stress and dehydration [28]. Transgenic plants have shown better drought tolerance with the overexpression of *ANAC055* [29]. Similarly, rice showed tolerance to oxidative stress, high temperature and drought due to overexpression *SNAC3* whereas its suppression by RNAi resulted in enhanced sensitivities to these stresses [30]. Seventeen NAC genes of apple (*Malus domestica*) had differential expression against abiotic stresses (high salinity, ABA, drought and temperature) [31]. Similarly in maize, three *ZmNAC* (*ZmNAC18*, *ZmNAC51*, and *ZmNAC145*) had up and down-regulated expression in drought tolerant and susceptible genotypes [32].

Like abiotic stresses, biotic stresses also induce the expression of NAC genes. In *Solanum tuberosum*, 44 *StSAP* had the differential expression against DL--amino-n-butyricacid (BABA), acibenzolar-s-methyl (BTH), and *Phytophthora infestans* inoculum (Pi isolate US8:Pi02-007) stresses [33]. *SISRN1*, an NAC TF, positively regulates the defense against *Botrytis cinerea* and *Pseudomonas syringae* pv. tomato DC3000 in tomato [34]. It also plays as positive role against drought and oxidative stress. That indicates the role of some NACs as positive regulators in both biotic and abiotic stresses. Three *FvNAC* had up-regulated expression during infection with *C. gloeosporioides* whereas six *FvNAC* had down-regulated expression when infected with *R. solanacearum* [35].

NACs play an important role in the regulation of the fruit development in plants. *SlNAC4* of tomato have been previously identified as the positive regulator of the fruit ripening in tomato [36]. Tomato *SlNAC1* gene has been found to be involved in the fruit pigment alteration and fruit softening in both abscisic acid-dependent and ethylene-dependent pathways [37]. Similarly, NOR is an NAC TF involved in delaying the fruit ripening in the tomato [38]. *FcNAC1* of strawberry (*Fragaria chiloensis*) has a role in the fruit softening via pectin regulation [39]. In *Cucumis sativus*, there are 12 NAC that are involved in the fruit development as these are the targets of the 13 mico-RNAs [40]. *AtNAP*, an NAC transcription factor in *A. thaliana*, has a role in the fruit senescence as it is accumulated during the fruit maturation [41]. It has been found that *CitNAC*, which is similar to *AtNAP*, is expressed in the pulp and peel of the orange during the fruit development [42].

Almonds (*Prunus dulcis*) are considered nuts from which edible seed is used as a commercial product. Almonds originated from Central Asia [43]. It is a widely cultivated crop in the United States, Spain, Italy, Turkey, Syria, and Iran [44]. Almond is the earliest deciduous nut tree that blooms in the spring due to low winter chilling requirements and quick growth response to warm temperatures [45]. Almond nuts contain essential nutrients including vitamins, minerals, proteins, amino acids, carbohydrates, fatty acids, and lipids, along with other secondary metabolites [46]. Genetic and environmental factors also have a major impact on the nutritional quality of the almond fruit.

NAC TFs have been studied in many plant species, including *A. thaliana* [11], rice [6], maize [47], soybean [48] and potato [33]. Almond NAC have not been systemically analysed by any study at genomic level. So given the vitality of NAC TFs, this study can help in a comprehensive understanding of NACs in almond. In this article, we identified the NAC genes in almonds and reported chromosomal positions, gene structure, phylogenetic analysis, gene duplication and syntenic analysis to find homology with *A. thaliana* and rice NAC TFs. Differential genes expression was also performed using RNAseq data to determine the differential expression of NAC genes in normally and abnormally grown almonds at various periodic developmental stages after flowering. This study will help in understanding the structural and functional analysis of NAC TFs in almonds.

## 2. Results

### 2.1. Identification of PdNAC Gene Family

BLASTp and domain analyses resulted in the identification of 106 NAC genes in *P. dulcis* after removal of redundant sequences having 100% similarity. These peptide sequences contained NAM domain family (PF02365) as protein sequences have been given in Supplementary Data S1. Identified genes were renamed from *PdNAC1* to *PdNAC106* according to their chromosomal position starting from chromosome 1. Three genes were not annotated on any of *P. dulcis* chromosomes, these were renamed from *PdNAC104*, *PdNAC105*, and *PdNAC106*. Detailed information of identified NAC of almond is represented in the Supplementary Table S1. Peptide sequences of *PdNAC* ranged from 155 AA residues (*PdNAC80*) to 891 AA residues of (*PdNAC29*).

### 2.2. Chromosomal Mapping and Cis-Acting Regulatory Analysis of NAC Genes

**Figure 1:**
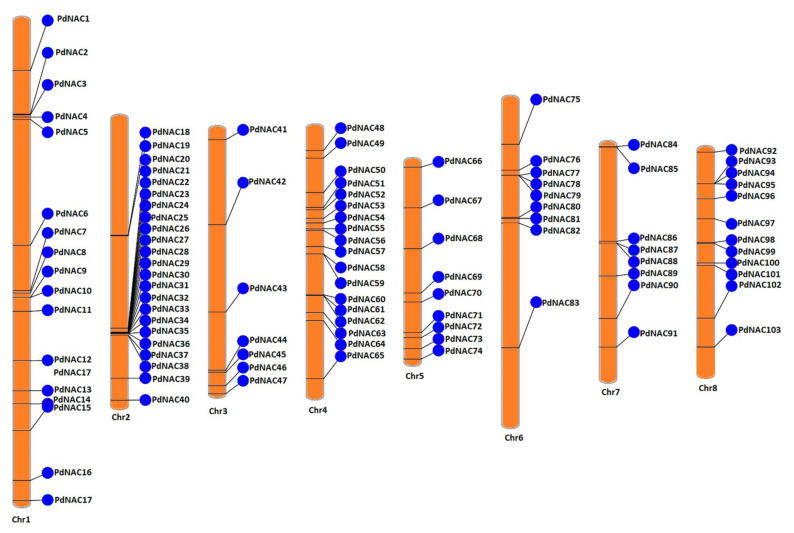
Chromosomal mapping of *PdNAC* genes. *PdNAC* genes have been predicted on the chromosomes of *P. dulcis*. Chromosome numbers is present below each chromosome.

Phenogram analysis of 103 *PdNAC* genes revealed that these are widely dispersed on all chromosomes of *P*. *dulcis* as shown in Figure 1. However, three genes were not annotated on the chromosomes, so their positions are not detected in the phenogram. Chromosomes 1, 2, 3, 4, 5, 6, 7, and 8 contained 17, 23, 7, 18, 9, 9, 8, and 12 *PdNAC* genes, respectively. Chromosome 2 contained the highest number of genes whereas chromosomes 3 contained the least number of *PdNAC* genes. *PdNAC2* and *PdNAC3* were clustered on the chromosome 1. From *PdNAC21* to *PdNAC36*, sixteen *PdNAC* genes were clustered on the chromosome 2. On chromosome 4, *PdNAC58*, *PdNAC59* and *PdNAC60*, *PdNAC61*, *PdNAC62* were clustered. *PdNAC77*, *PdNAC78*, and *PdNAC79* were clustered on chromosome 6. *PdNAC84* and *PdNAC85* were clustered on the chromosome 7 along with another cluster of *PdNAC87* and *PdNAC88*. However, chromosome 8 had only one cluster having *PdNAC93*, *PdNAC94*, and *PdNAC95*.

**Figure 2:**
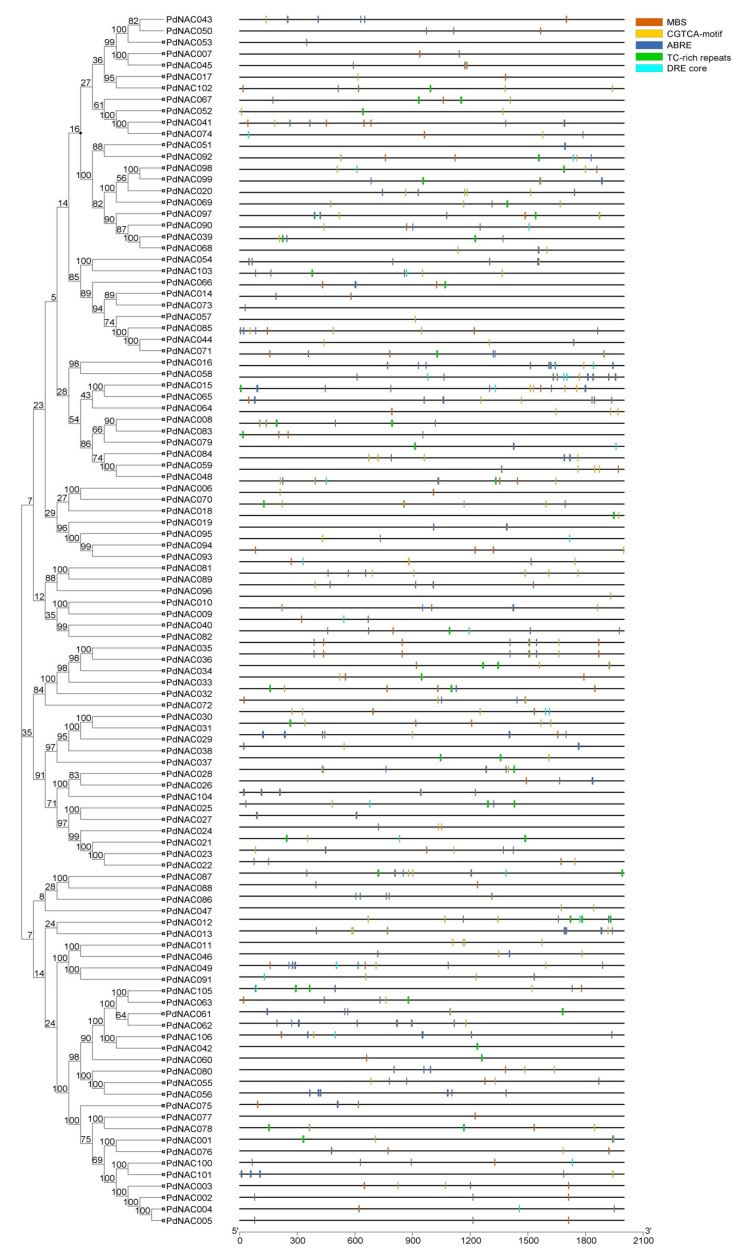
Prediction of *cis*-regulatory elements of *PdNAC*. *Cis*-regulatory elements are shown in upstream region of the *PdNAC*. *Cis*-regulatory elements are shown in different colors.

Stress-related five *cis*-elements ABRE, DRE, CGTCA, TC-rich, and MBS were selected and promoter regions of (2000 bp upstream of *PdNAC* genes) were scanned for the presence of these elements as represented in Figure 2. ABRE elements were most frequent and were present in 85 *PdNAC* genes (Supplementary Data S2). The highest number of ABRE elements were present in *PdNAC15* which contained 13 ABRE elements. CGTCA-motif was present in 80 genes with *PdNAC89* containing the most. DRE was present only in 27 genes as it is induced under osmotic and drought stress. Among all, DRE was the least occurring in *PdNAC* and *PdNAC15* contained 3 DRE elements. Whereas 64 *PdNAC* had 91 MBS elements from which 4 MBS sites were present in the *PdNAC41*. MBS is a MYB binding site responsible for drought inducibility. TC-rich elements were detected in the 37 genes that are responsible for defence and stress-responsiveness.

### 2.3. Phylogenetic Analysis of PdNAC and AtNAC

**Figure 3:**
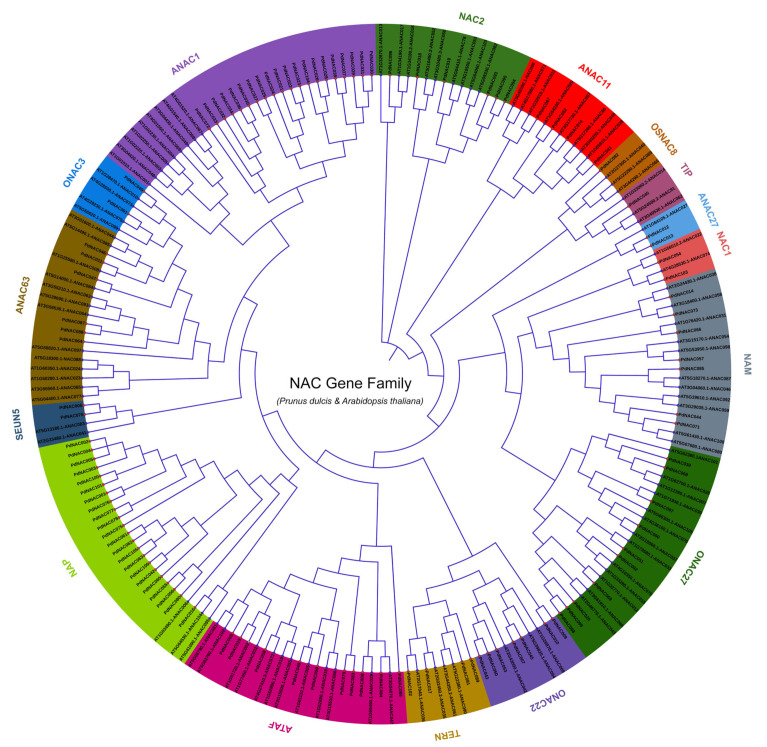
Phylogenetic analysis of *P*. *dulcis* and *A*. *thaliana* NAC. *PdNAC* and *AtNAC* are divided into fourteen subgroups according to the subgrouping of *A. thaliana.*

The peptide sequences of *A. thaliana* and *P. dulcis* were used for the construct of the unrooted phylogenetic tree to explore the evolutionary relationship. Based on the homology of NAC proteins in *Arabidopsis*, 106 peptide sequences of *P. dulcis* were divided into sixteen groups as shown in Figure 3. *P. dulcis* NAC representatives were present in NAC2 (6), ANAC11 (4), OsNAC8 (1), TIP (1), ANAC27 (2), NAC1 (2), NAM (7), ONAC27 (10), ONAC22 (7), TERN (4), ATAF (10), NAP (22), SEUN5 (2), ANAC63 (6), ONAC3 (2), and ANAC1 (20) subgroups. TIP and OsNAC8 subgroups contained only 1 NAC that was *PdNAC40* whereas NAP subgroup contained the highest number of NAC genes that were 22. The phylogenetic analysis revealed the functional diversity of NAC in *P. dulcis* which was consistent with the *A. thaliana* NAC.

Phylogenetic analysis of NAC genes of *P. dulcis, V. vinifera*, *P. persica* and *A. thaliana* showed that *P. dulcis* NACs are closely related to the NACs of *P. persica* as shown in Supplementary Figure S1. None of *P. dulcis* NAC had a close relationship with NACs of *V. vinifera* and *A. thaliana*. However, subgroups 5, 6, and 8 contained only NACs of *P. dulcis* and *P. persica*.

### 2.4. Gene Structure, Motif Composition and Domain Analyses of PdNAC

**Figure 4:**
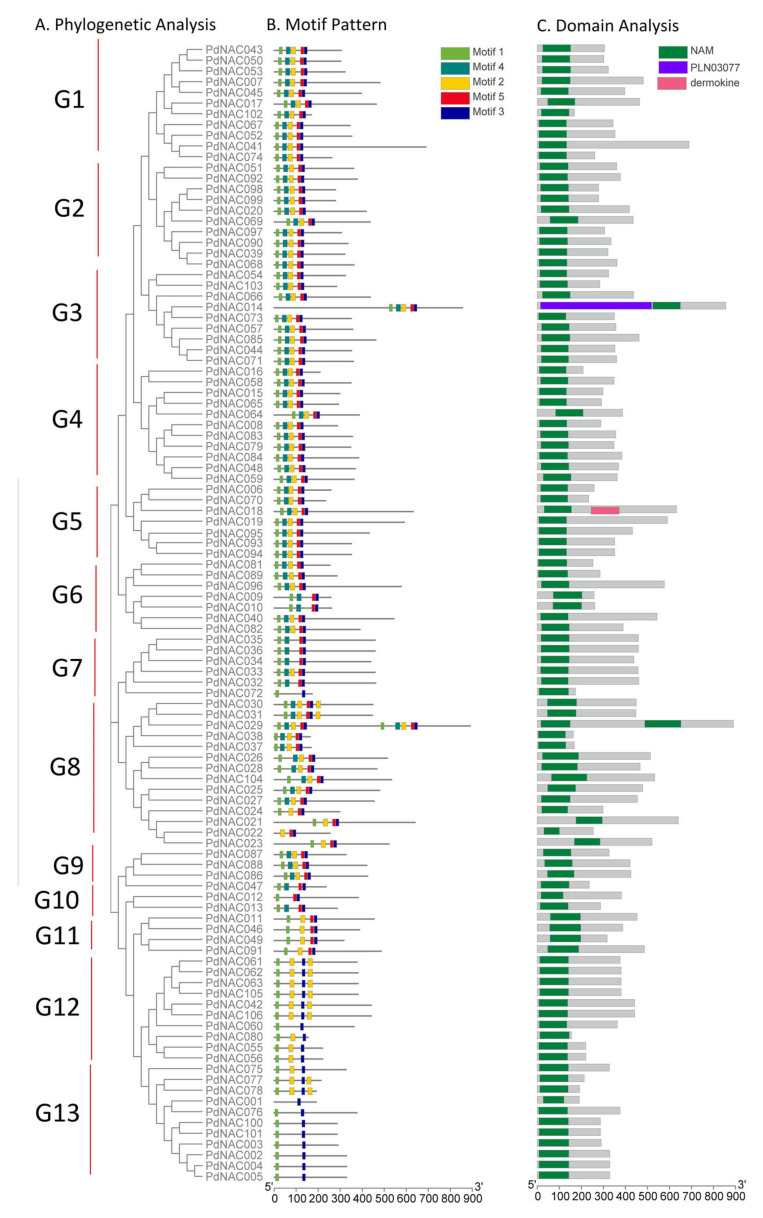
Gene structure and motif analysis of PdNAC. (**A**) Phylogenetic tree, (**B**) motif pattern, and (**C**) domain presence. Gene structure shows the presence of introns and exons in the genes.

Phylogenetic tree of 106 peptide sequences of *P*. *dulcis* was constructed and the tree consisted of thirteen subgroups as represented in Figure 4A. Largest subgroup, G8, contained 14 members of *P*. *dulcis* NAC. Whereas G10 contained only *PdNAC12* and *PdNAC13*. In all *P*. *dulcis* NAC, NAM domain was conserved as predicted by conserved domain database (CDD) as shown in Figure 4C.

**Figure 5:**
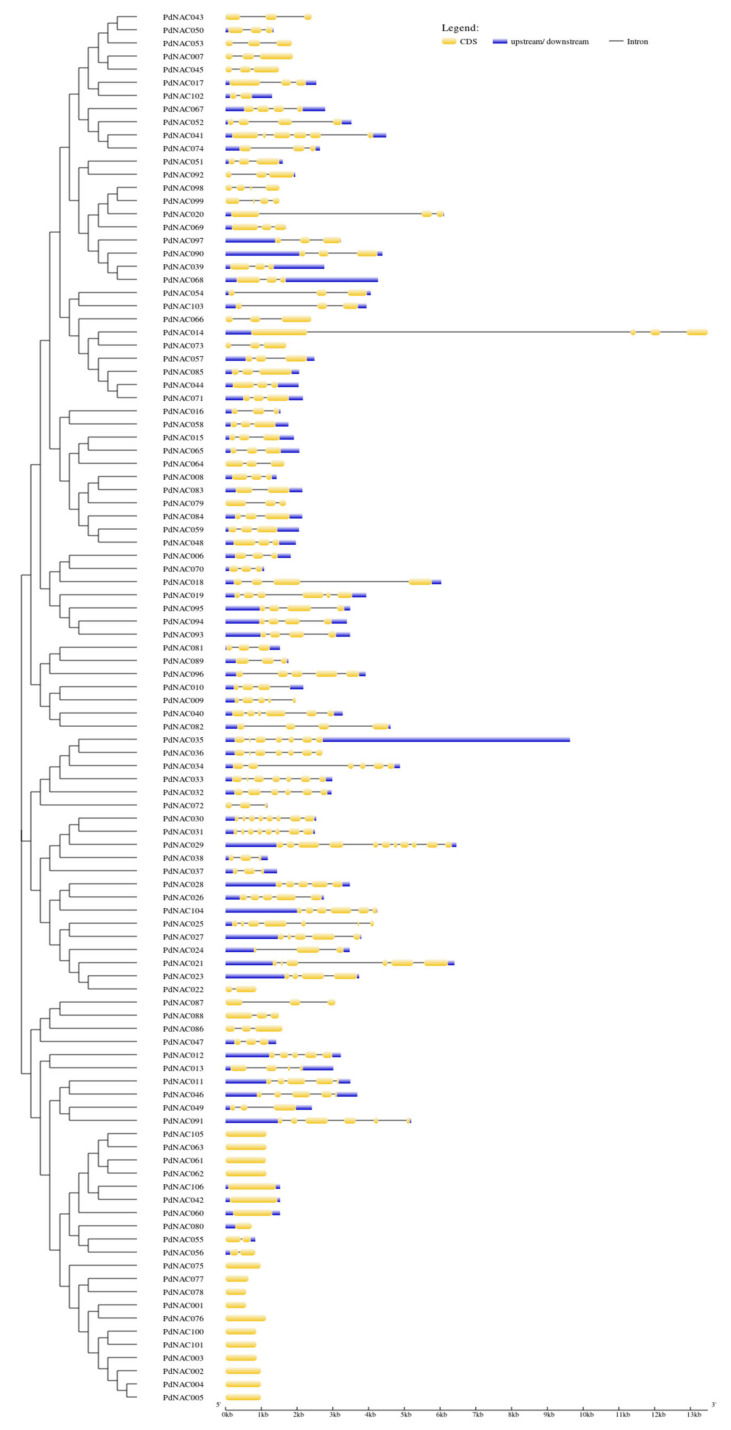
Gene structure prediction of *PdNAC* genes: Gene structure of *PdNAC* genes is shown in the figure where CDS, UTRs and intronic regions of the genes are represented.

Gene structure of *PdNAC* genes is shown in the Figure 5. In most of the *PdNAC* sequences, exons were interrupted by the introns. Some sequences contained the UTRs at 5′ and 3′ ends of the genes. *PdNAC29* contained 10 introns that were highest in numbers among all *PdNAC*. However, some lacked intronic regions in their ORFs, as member of subgroups 12 and 13 lacked the intronic regions and consisted of single exons. Interestingly, members of same subgroups had a similar exon and intron pattern regarding exon length, intron phase and intron number.

Further, motif analysis of 106 *PdNAC* proteins was carried out using MEME web server as shown in Figure 4B. Motif’s composition and pattern is shown in Supplementary Figure S2. Five motifs were predicted in the *PdNAC* genes. However, a similar pattern was observed in the members of the same subgroup. All five motifs were present from subgroup G1 to G5. However, subgroup G6-G9 had a variable motif pattern. Some members of these clades contained all five motifs whereas others contained four motifs. However, in *PdNAC72* belonging to G7, only two motifs were conserved. G10 had only two members *PdNAC12* and *PdNAC13*, each containing 3 and 4 conserved motifs, respectively. Subgroups G11, G12, and G13 lacked Motif 4. In G13, motif pattern was variable as sequences contained single, two or three motifs.

### 2.5. Gene Duplication and Syntenic Analyses of P. dulcis NAC Genes

**Figure 6:**
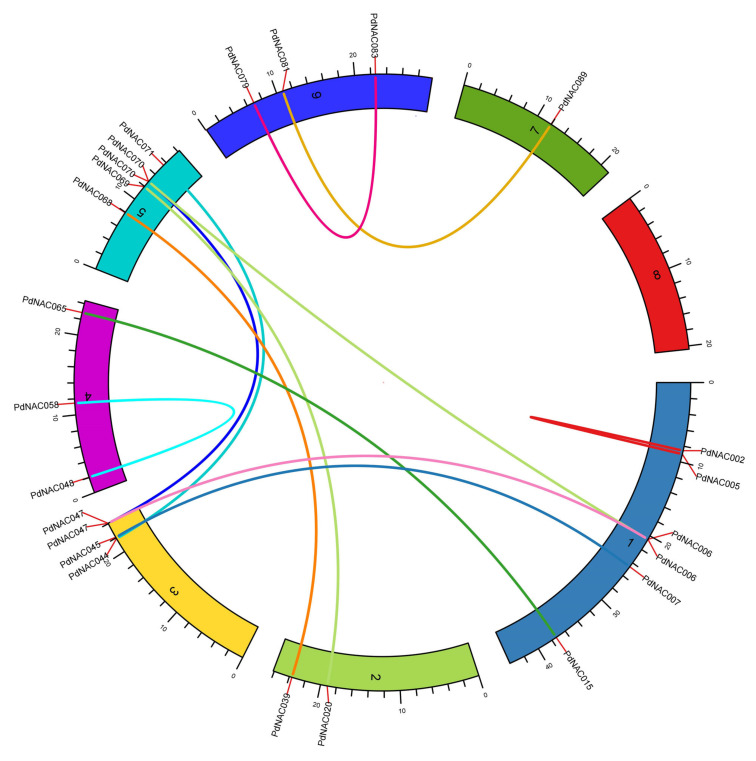
Gene duplication in *PdNAC***.** In all, 12 pairs of the genes were duplicated in the *PdNAC* genes. These genes were located on different chromosomes. Chromosome 8 had no duplicated gene whereas all other contain duplicated genes.

MCScanX revealed 2136 duplicated blocks in the genome of *P*. *dulcis*. Total 12 pairs of the duplicated genes were predicted in 106 NAC of *P*. *dulcis* as represented in Figure 6. These duplicated genes were most common on the chromosome 1. It was followed by the chromosome 5 that contained 5 duplicated genes. However, chromosome 8 did not contain any duplicated gene.

**Figure 7:**
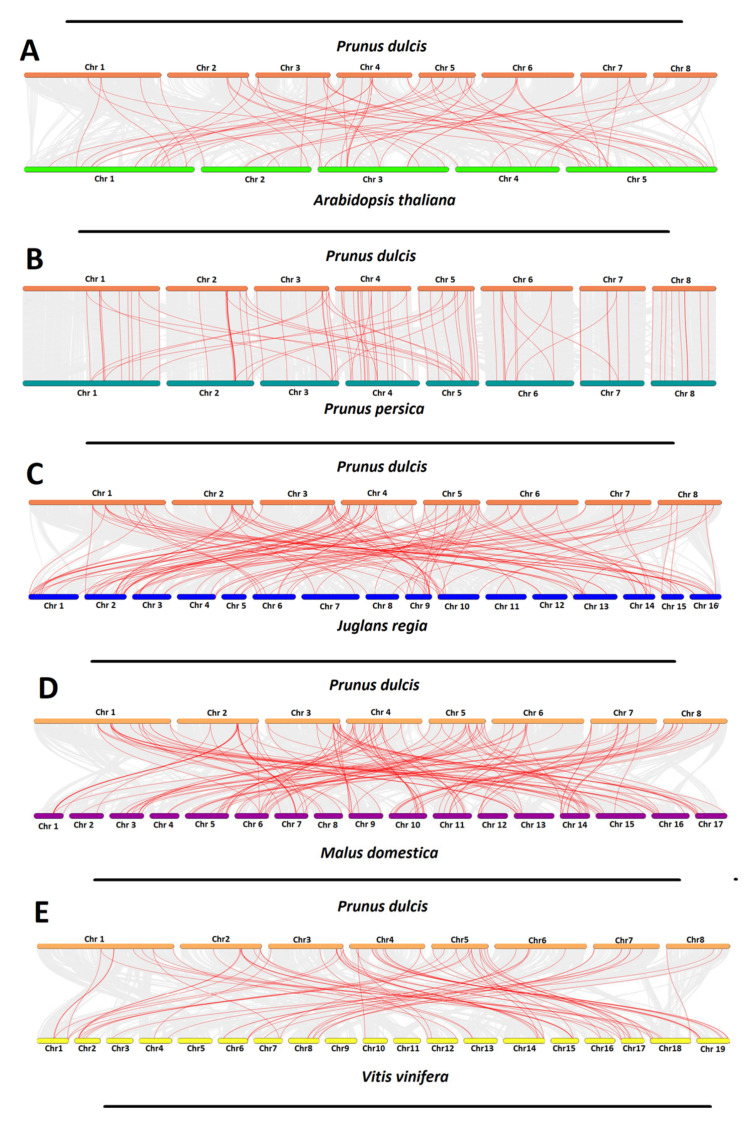
Visualization of the syntenic analysis. Synteny of the *P. dulcis* NAC has been visualized with the NAC of *A. thaliana* (**A**), *P. persica* (**B**), *J. regia* (**C**), *M. domestica* (**D**), and *V. vinifera* (**E**). Red lines between the genomes show the synteny between the genes.

**Figure 8:**
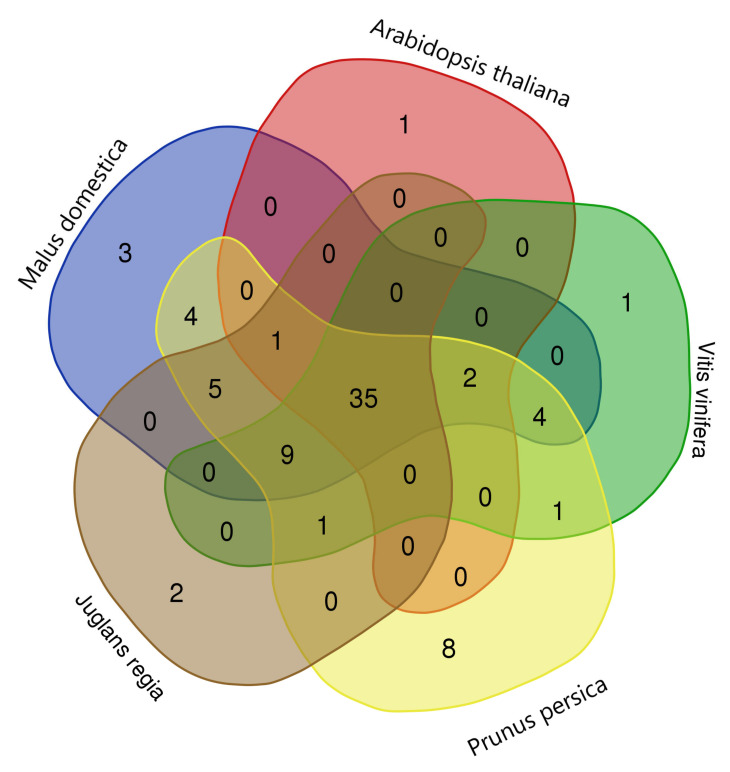
Venn diagram of syntenic analysis. The diagram shows the unique and common *PdNAC* having synteny with *A. thaliana*, *P. persica*, *J. regia*, *V. vinifera* and *M. domestica*.

Syntenic analysis of *P. dulcis* genes were performed with the NAC genes of *A. thaliana*, *P. persica*, *J. regia*, and *M. domestica* to identify the homologous pairs as shown in Figure 7. A total of 76 NAC genes of *P. dulcis* had syntenic relationship with the NAC genes of *A. thaliana* (58), *P. persica* (71), *J. regia* (90), *V. vinifera* (53), and *M. domestica* (107) as shown in Supplementary Table S2. Some genes had multiple syntenic relationships with the genes of other closely related species. So, A total number of 64, 91, 125, 71, and 138, NAC genes of *A. thaliana*, *P. persica*, *J. regia*, *V. vinifera*, and *M. domestica*, respectively, had synteny with 76 *PdNAC* genes. Further, it was observed that 35 NAC genes of *P. dulcis* had homologues in all four compared organisms as shown in Figure 8. However, five *PdNAC* genes had common homologous in *M. domestica*, *P. persica*, and *J. regia* but had no syntenic homologue in *A. thaliana* and *V. vinifera*. Similarly, *V. vinifera*, *M. domestica*, and *P. persica* had homologues 4 *PdNAC* that lacked homology with other compared organisms. Whereas *A. thaliana*, *P. persica*, *J. regia*, *V. vinifera* and *M. domestica* each had unique homologues of 1, 8, 2, 1, and 3 *PdNAC* genes, respectively.

### 2.6. Physicochemical Properties and Subcellular Localization Prediction

**Figure 9:**
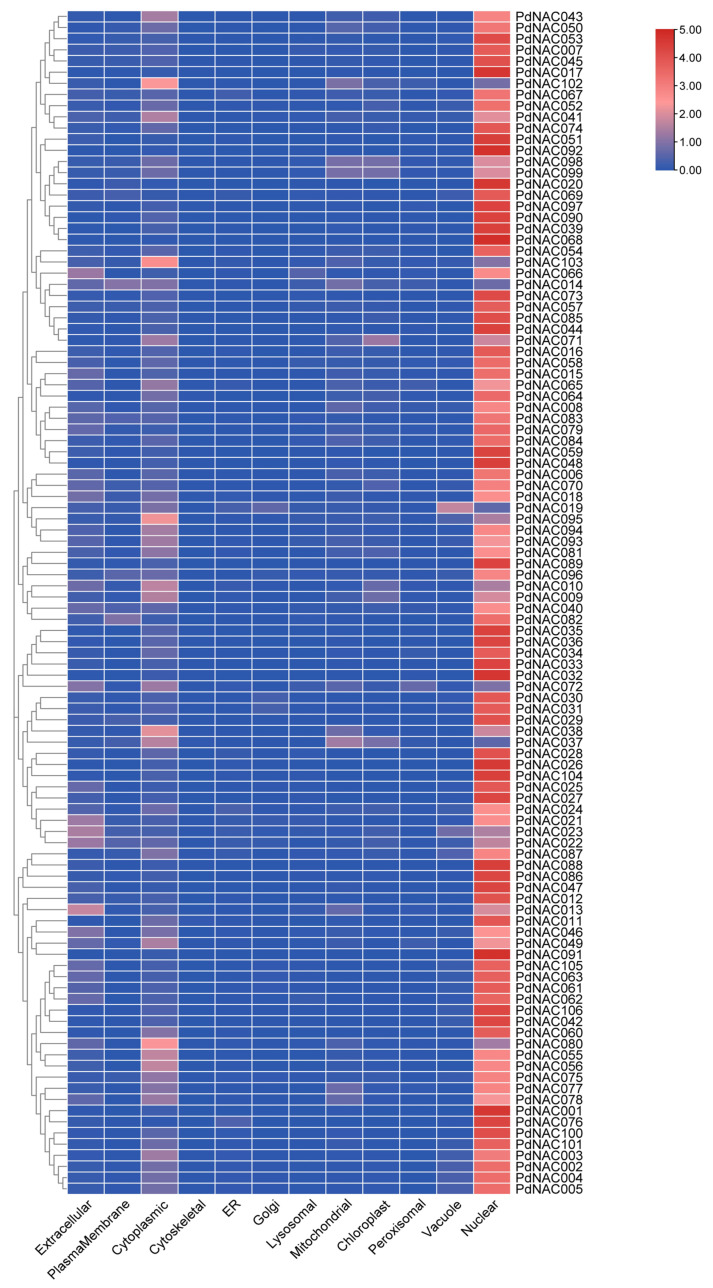
Subcellular localization prediction of *PdNAC*. Subcellular localization of the *PdNAC* is shown along with the phylogenetic tree of *PdNAC*. Majority of the *PdNAC* are localized in the nucleus.

Molecular weight (MW) of *P. dulcis* NAC genes varied from 17.98 kDa of *PdNAC80* to 101.73 kDa of *PdNAC29* as represented in Supplementary Table S1. Isoelectric point (PI) of *PdNAC* varied between 4.53 of *PdNAC19* and 9.45 of *PdNAC13*. Most of the *P. dulcis* NAC proteins were predicted to be localized in the nucleus whereas 6 and 3 NAC were localized in the cytoplasm and extracellular space as shown in Figure 9.

### 2.7. Gene Ontology Analysis

Gene ontology analysis predicted the biological processes, molecular functions, and cellular compartments *P. dulcis* NAC genes as shown in Figure 9 whereas gene ontology table is provided in Supplementary Table S3. Gene annotation predicted that majority of *P. dulcis* genes were involved in regulation of transcription indicating their role as transcription factor in *P. dulcis*. Their role in other biological processes included translation, system development, plant-based secondary cell wall genesis, fruit dehiscence and response to wounding. Whereas most of *PdNAC* were integral membrane components while others were predicted to be compartmentalized in the nucleus and ribosomes. Study of molecular functions revealed that *PdNAC* were involved in DNA binding, hydrolase binding and kinase binding activity.

### 2.8. Differential Gene Expression of NAC Genes in Normal and Abnormal Fruitlets

**Figure 10:**
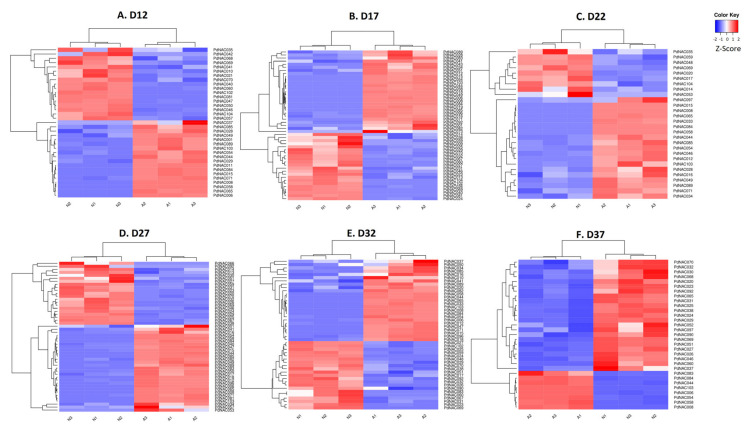
Differential gene expression. Heatmaps of differential gene expression of *PdNAC* in almond fruit is shown at developmental stage D12 (**A**), D17 (**B**), D22 (**C**), D27 (**D**), D32 (**E**), and D37 (**F**). Heatmaps are based on Z-score.

RNA-seq analyses of Normal and Abnormal fruitlets during their developmental stage at day 12, 17, 22, 27, 32, and 37 were carried out to determine the differential expression pattern. Z-score of differentially expressed *PdNAC* at all six developmental stages are shown in Figure 10. Z-score of differentially expressed *PdNAC* genes have been provided in Supplementary Table S4. DeSeq2 revealed the up and down regulation of gene in the normal versus abnormal fruitlets. At D12, 17 *PdNAC* genes were up-regulated whereas 18 were down-regulated. Similarly, 22 NAC genes were up-regulated and 27 were down-regulated at D17. At D22, a total of 9 and 19 NAC genes had up and down-regulated expression, respectively. A total of 21 and 29 *PdNAC* were up and down-regulated at developmental day of 27, respectively. At the 32 day of development, expression of 21 and 25 *PdNAC* genes was up and down-regulated, respectively. Finally, at 37 day of fruitlet’s development, 23 and 8 *P. dulcis* NAC genes were up-regulated and down-regulated, respectively. *PdNAC69* had up-regulated expression in fruitlets at all developmental stages. Whereas *PdNAC8*, *PdNAC44*, *PdNAC54*, *PdNAC58*, *PdNAC84*, and *PdNAC103* were down-regulated in fruitlets of all sampled growth stages. To observe the difference of genes expression between the normal and abnormal fruit development, volcano plots were generated as shown in the Supplementary Figure S4. Whereas Supplementary Figures S5–S10 are given for significant DGE for each sampling stage. There were 7 genes that were present at all the stages having significant expression that are *PdNAC8*, *PdNAC44*, *PdNAC54*, *PdNAC58*, *PdNAC69*, *PdNAC84*, and *PdNAC103*. Significantly expressed NAC genes common to various stages have been represented in the Supplementary Table S5.

## 3. Discussion

Almonds are dry fruits that are used worldwide owing to their nutrition. Given their importance, NAC TFs play a major role in the development of the almond fruits, secondary wall synthesis and response to biotic and abiotic responses [10,49]. NAC genes have been known to enhance salt tolerance and drought resistance in rice [22]. With the development and easier accessibility of genome sequencing techniques, NAC genes have been identified in a number of organisms. Until now, genome wide analysis of NAC genes in *P. dulcis* has not been performed. Therefore, this genome wide study has identified NAC genes in *P. dulcis*, performed detailed analysis and analysed their differential expression using RNA-seq data.

The number of NAC genes in different organism varies as there are more than 100 in most of the organisms [31]. A total of105 and 104 NAC genes have been identified in *A. thaliana* and *Solanum lycopersicum* [7,50]. In this study, 106 NAC genes have been identified in *P. dulcis*. In *P. dulcis*, NAC genes are unevenly distributed on the chromosomes. There are sixteen *PdNAC* genes that were clustered on chromosome 2, whereas others *PdNAC* genes were either spread out or clustered in the form of two or three genes on the chromosomes. Similarly, clustered NAC genes have been found in the genome of *Theobroma cacao* [51]. ABA responsive element (ABRE), *cis*-regulatory elements are abundant in the upstream promoter regions of *PdNAC* genes as in maize NAC [47]. Similarly, ABRE abundance has also been found in the *SNAC1* in the rice [52]. ABA-responsive elements are involved in the drought stress whereas MYB binding site (MBS) is responsible for the drought inducibility in the plants.

The phylogenetic tree of *PdNAC* genes was divided into the sixteen subgroups in accordance to the *A. thaliana* and *T. cacao* NAC phylogeny [7,51]. In *A. thaliana*, there were 13 subgroups whereas in *T. cacao*, there were 17 subgroups along with one unknown group. There was no unknown subgroup in the *P. dulcis* as all fourteen subgroups had similar members in the *A. thaliana* and *T. cacao*. However, in maize, NAC were divided into thirteen subgroups. Phylogenetic analysis revealed isolation of different types of the NAC TFs in *P. dulcis*. The most abundant proteins were present in NAP and *ANAC01* subgroups indicating their importance of these two subfamilies of NAC TFs in *P. dulcis*. Similarly, NACs of *P. dulcis* had a close relationship with the NACs of *P. persica*. Whereas none of *P. dulcis* NAC had a closer relationship with the NACs of *A. thaliana* and *V. vinifera*. *A. thaliana* was more distantly related with NACs of *P. dulcis* than the *V. vinifera* NACs. However, few NACs of *P. persica* had closer relationship with the NACs of *V. vinifera*. Furthermore, *A. thaliana* NACs were distantly rooted.

Five motifs were identified in the *P. dulcis* genome that had a pattern according to the genes position in the phylogenetic tree. This pattern was well observed in the *S. lycopersicum* and *Solanum tuberosum* [33,53]. Gene structure analyses revealed that intronic regions were present, but the number of introns were variable according to *PdNAC* genes position in the phylogenetic subgroups. As in *T. cacao*, some of *PdNAC* did not have intronic regions and also lacked untranslated regions (UTRs) in their gene structure [51]. Intronless genes have major function in the basic cellular processes such as growth regulation, transcription, and immune response [54]. As *PdNAC* are involved in the transcription regulation, these can induce facilitative transcription regulation [55].

Gene duplication revealed that 12 *PdNAC* genes pair were duplicated. Gene duplication is important for the rapid evolution and expansion of the gene families as gene duplication events are found in various plants species. Like *P. dulcis*, 13 pairs of *FtNAC* genes were duplicated in the *Fagopyrum tataricum* [56]. *P. dulcis* NAC genes showed more synteny with the *J. regia* and *M. domestica* as compared to *P. persica*. However, *P. persica* is a closed relative of Almonds than *M. domestica* and *J. regia*. *P. dulcis*, *P. persica*, and *M. domestica* belong to the same family Rosaceae whereas *J. regia and A. thaliana* belongs to *Juglandaceae* and *Brassicaceae* families, respectively. Similarly, *V. vinifera* belonged to the Vitaceae family, therefore, it had 73 syntenic relations with *P. dulcis* NAC genes. *P. dulcis* had higher synteny with the *M. domestica* because later contains 180 NAC genes [31]. Multiple *MdNAC* have homology with single *PdNAC*. *PdNAC* were predicted to be localized in the nucleus for most of the *PdNAC*, however, some were localized in the cytoplasm and extracellular like in *T. cacao* [51]. Gene ontology analysis provided insights into *PdNAC*’s role in the biological process as transcription regulator and the function in the DNA binding. It also confirmed the subcellular localization of the *PdNAC* in the *P. dulcis* as these are concentrated in the nucleus whereas few are localized in cytoplasm and extracellular.

Differential gene expression using RNA-seq is a technique to determine the expression of genes using cDNA of the sample. In this study, SRA of developmental transcriptome profiling of normal and abnormal almond fruitlets at 12, 17, 22, 27, 32, and 37 days of development were used to determine the expression of *PdNAC* genes. RNA-seq analysis of NAC genes revealed the differential expression at various stages of the development. Some of the genes like *PdNAC69* had up-regulated expression whereas *PdNAC8*, *PdNAC44*, *PdNAC54*, *PdNAC58*, *PdNAC84*, and *PdNAC103* had down-regulated expression at all stages of the development. This indicates these genes had a constant expression at all tested stages. However, up and down-regulation of other genes varied with the day of development and growth stages. Nine *PdNAC*, *PdNAC23*, *PdNAC24*, *PdNAC25*, *PdNAC26*, *PdNAC27*, *PdNAC29*, *PdNAC31*, *PdNAC32*, and *PdNAC38* had up-regulated expression at day 17, 27, 32 and 37 of development, respectively. Whereas *PdNAC6* had downregulated expression at all these four stages.

Like *P. dulcis*, it has been found that 13 NAC genes had differential expression in numerous tissues during fruit growth and ripening in *M. domestica* as it has been shown that NAC regulate the pome development by ethylene dependent and independent manner [57]. Similarly, six strawberry NAC were involved in the regulation of fruit development and ripening [58]. In *Prunus sibirica*, eight *PsNAC* were involved in the fruit ripening whereas four *PsNAC* were responsible for the hardening and maturation of kernel, each. In *P. persica*, 16 genes having differential expression were annotated as NAC and MADS-box genes during the fruit development [59]. Like these organisms, *PdNAC* also had differential gene expression at various developmental stages of the fruits in *P. dulcis*. At different days of development, NAC had variable expression whereas some had constant up-regulated and down-regulated expression at all stages of fruit development in normal versus abnormal fruit development. So, this study provides detailed analysis on the *P. dulcis* NAC and determines their expression during the fruit development in *P. dulcis*.

## 4. Materials and Methods

### 4.1. Identification of PdNAC Gene Family

Protein FASTA file of almond (*P*. *dulcis*) of latest genome assembly (GCF_902201215.1) was retrieved from NCBI (accessed on 15 July 2021). Further, peptide sequences of annotated NAC genes of *A. thaliana* were retrieved from PlantTFDB (http://planttfdb.gao-lab.org/, accessed on 15 July 2021). A local BLASTp was performed using BioEdit software with BLOSUM62 matrix and 10 as the expectation value (E value) against almond peptide FASTA file using as local database for BLAST search. NAC peptide sequences of *A*. *thaliana* were used as query for BLASTp. BLASTp output was checked for the presence of NAM domain (Pfam:PF02365). Those sequences containing the NAM domain were selected, and redundant sequences having 100% similarity were removed. Putative NAC genes were renamed according to their distribution on chromosomes.

### 4.2. Chromosomal Mapping and Cis-Acting Regulatory Analysis of NAC Genes

Chromosomal position of the NAC genes was predicted using phenogram web server (http://visualization.ritchielab.org/phenograms/plot, accessed on 20 July 2021). Phenogram creates ideograms with different colors with exact position of genes [60].

For *cis*-acting regulatory analysis, 2000 bp of genomic sequence upstream of NAC genes were retrieved. These sequences were used in the PlantCARE database (http://bioinformatics.psb.ugent.be/webtools/plantcare/html/, accessed on 1 September 2021) to predict *cis*-regulatory elements. TB tools was used to visualize the *cis*-acting regulatory regions in upstream sequences.

### 4.3. Gene Structure and Motif Analyses of PdNAC

To analyze the gene structure of the NAC genes, Coding DNA Sequences (CDS) and gene sequences were used in Gene Structure Display Server (http://gsds.gao-lab.org/, accessed on 12 September 2021). Phylogenetic tree was generated using Neighbour-Joining method with p-distance and 1000 bootstraps in MegaX-V10.2.4. Phylogenetic tree was generated to visualize the tree along with the motifs and gene structure for better understanding of motifs and structure distribution across the subgroups. Motifs were predicted using MEME web server (https://meme-suite.org/meme/, accessed on 1 September 2021). Protein domains were predicted using conserved domain database (https://www.ncbi.nlm.nih.gov/Structure/cdd/wrpsb.cgi, accessed on 1 September 2021). These output files were used in TBTools to generate and visualize gene structure of the NAC genes [61].

### 4.4. Physicochemical Properties and Subcellular Localization of PdNAC

Physicochemical properties were predicted using the ProtParam in Expasy web server (https://web.expasy.org/protparam/, accessed on 1 September 2021). ProtParam computationally calculates physicochemical properties (Molecular weight and isoelectric points) for a given sequence of proteins. Subcellular localization of NAC proteins was predicted using Cello (http://cello.life.nctu.edu.tw/, accessed on 1 September 2021) and WoLF PSORT (https://wolfpsort.hgc.jp/, accessed on 1 September 2021). WoLF PSORT predicts proteins subcellular localization on the basis of correlative sequence features and sorting signal motifs in protein sequences [62]. Whereas Cello uses homology search method and two level support vector machine (SVM) to predict the sub-cellular localization using protein sequences [63].

### 4.5. Multiple Sequence Alignment and Phylogenetic Analysis of PdNAC

NAC peptide sequences of *A. thaliana*, rice, and almonds were pooled together in a FASTA file. ClustalW-V2.1 was used to perform multiple sequence alignment (MSA) of NAC sequences on galaxy webserver [64,65]. Multiple sequence alignment output was used for the construction of the phylogenetic tree using FastTree2 in galaxy web server [66]. FastTree2 uses Maximum-Likelihood method for the construction of phylogenetic tree of hundreds of thousands of protein sequences in a time efficient manner. Jones-Taylor-Thornton 1992 model (JTT) and constant rates (CAT) were used as evolutionary models for phylogenetic tree. Finally, phylogenetic tree was edited in iTOL tool (https://itol.embl.de/, accessed on 11 August 2021).

Furthermore, NAC genes from *V. vinifera*, *A. thaliana*, *P. persica*, and *P. dulcis* were used to generate a phylogenetic analysis to determine the closer relationship of *PdNAC* genes with these organisms.

### 4.6. Analysis of Gene Ontology

*PdNAC* genes annotation was carried out using Blast2Go [67]. In Blast2Go, biological activity, molecular functions and cellular compartments of the NAC genes were determined. Blast2Go performs BLASTx, InterPro Scan, mapping and annotation using transcript sequences. This analysis was carried out using the default settings of Blast2Go.

### 4.7. Gene Duplication and Synteny Analyses

Gene duplications in NAC genes were predicted using MCScanX with default parameters [68]. MCScanX uses BLASTp output results to calculate the duplications events in a genome. These duplicated genes were visualized in a circos form using TB tool. One-Step MCScanX was used to predict the synteny between NACs of almonds with the NAC of *A*. *thaliana*, *Prunus persica* (Peach), *Juglans regia* (Walnut), and *Malus domestica* (Apple) using genome feature files (.gff) and genomic FASTA files. Dual synteny plotter was used to visualize the synteny. TB tools was used for the last two steps [61]. Homologous genes from the synteny analysis were used to build Venn diagram (http://bioinformatics.psb.ugent.be/webtools/Venn/, accessed on 13 September 2021) to further analyse the distribution of the *P. dulcis* NAC genes with the compared organisms containing orthologous NAC genes.

### 4.8. Differential Expression Analysis of PdNAC in Fruits

RNA-sequencing data sequenced by Illumina Hiseq 2500 of Almond cultivar ZhiPi (BioSample: SAMN12855948) was used to analyze NAC’s expression during various stages of almond fruit development. For sampling, thirty almond trees were selected randomly based on phenotypic investigation [69]. Fruits having diapause atrophic growth were selected as abnormal fruits. Normal and abnormal growing fruits were sampled at six different times that are 12, 17, 22, 27, 32, and 37 days after flowering (DAF). Three replicates were selected for each normal and abnormal growing fruit at the above mentioned six stages of growth after flowering. Normal and abnormal fruits were sampled from the same branch. Sequence read archives (SRA) were retrieved from NCBI Geo Database (https://www.ncbi.nlm.nih.gov/sra, accessed on 10 August 2021). SRA and their accessions along with the condition as normal or abnormal are provided in Supplementary Table S6. SRA files were converted into FASTQ format to use them for RNAseq analysis [70]. FASTQ files generated the pair-end data containing forward and reverse reads from SRA files. FastQC was used to check the quality of the reads at each step [71]. Cutadapt was used to trim adapter and low-quality sequences from the reads [72]. Quality cutoff and minimum length of sequences was set at 20 to remove small and low-quality sequences. GTF files from almondV2 assembly (Refseq: GCF_902201215.1) was used as reference genome for the alignment of the reads in STAR [73]. Mapped reads information is represented in Supplementary Table S7. Number of reads per gene were determined using FeatureCounts [74]. DESeq2 was used for differential gene expression analysis of normal fruitlets and abnormal fruitlets at different days of their development as plots of DESeq2 have been shown in Supplementary Data S3 [75]. Normalization was carried out by DESeq2. Filtration of Differentially expressed genes was carried out at significant adjusted p-value > 0.05 and LogFC > 1 to filter out insignificantly expressed genes (Supplementary Table S8). Volcano plot was created using differential expression data from DESeq2. Differentially expressed NAC genes were labelled in the volcano plot.

Z-score of differentially expressed genes using normalized count was computed as

$$z_{i,j}=\frac{x_{i,j}-\bar{x_{i}}}{s_{i}}$$

where z-score *z_i,j_* for a gene *i* in a sample *j*, given the normalized count *x_i,j_* is computed with $x_{\bar{i}}$, the mean and *s_i_*, the standard deviation of the normalized counts for the gene *i* over all samples. These statistical analyses for all the samples were performed using Table Compute, a galaxy wrapper for Pandas Data Analysis Library. The heatmap of the differentially expressed genes was generated using heatmap2 module. These all analyses were performed using the Galaxy web server [65].

## 5. Conclusions

This study aims to provide detailed information on *Prunus dulcis* regarding their potential physiological role, associated molecular mechanisms and their expression during fruit development. Using bioinformatics and phylogenetic analysis, we identified 106 NAC genes in *P*. *dulcis*. The *PdNAC* gene’s chromosomal location, domains and conserved motifs were identified having a constant pattern according to their membership of phylogenetic subgroup. Further, *PdNAC* genes were analyzed for the gene duplication and their synteny with *Arabidopsis thaliana*, *Prunus persica*, *Malus domestica*, and *Juglans regia* revealed the orthologs in other species. Furthermore, subcellular localization and gene ontology predicted that *PdNAC* are localized in the nucleus and involved in DNA binding as transcription regulators. RNA-seq analyses of the *PdNAC*, at day 12, 17,22, 27, 32 and 37 of the normal and abnormal fruits development, revealed the up and down-regulated expression of these genes. It was found that three *PdNAC* genes had up-regulated and eight *PdNAC* genes had down-regulated expression at all growth stages of almond fruits. These systematic analyses of *PdNAC* will be helpful for the future studies to functionally characterize *PdNAC* genes and using them to improve the fruit development in almond varieties.

## Data Availability

The data presented in this study are available in insert article or Supplementary Material here.

## Supplementary Materials

The following are available online at https://www.mdpi.com/article/10.3390/plants10102200/s1, Figure S1. Phylogenetic tree of NAC genes of *At. thaliana*, *P. dulcis*, *P. persica*, and *V. vinifera*. Phylogenetic tree represents close relationship of *P. dulcis* NAC with *P. persica* and distant with other organism’s NACs. Figure S2. Motif’s pattern. Motif’s pattern and composition is shown. (A) Motif 1, (B) Motif 2, (C) Motif 3, (D) Motif 4, (E) Motif 5 are shown. Figure S3. Prediction of gene ontology of *PdNAC***.** Ontology analysis predicted *PdNAC* to be DNA binding transcription factors involved in the transcription regulation. It also predicted their subcellular localization. Biological processes (BP), cellular compartments (CC), and molecular function (MF) of *PdNAC* are visualized on *X*-axis whereas *Y*-axis contain the number of sequences. Figure S4. Volcano plots of *PdNAC*. Volcano plots of differentially expressed *PdNAC* genes are shown. Volcano plots of *PdNAC* DEG at (A) D12, (B) D17, (C) D22, (D) D27, (E) D32 and (F) D37 of the developments are shown. Figure S5. Differential gene expression. Heatmaps of differential gene expression of *PdNAC* in almond fruit is shown at developmental stage D12. Figure S6. Differential gene expression. Heatmaps of differential gene expression of *PdNAC* in almond fruit is shown at developmental stage D17. Figure S7. Differential gene expression. Heatmaps of differential gene expression of *PdNAC* in almond fruit is shown at developmental stage D22. Figure S8. Differential gene expression. Heatmaps of differential gene expression of *PdNAC* in almond fruit is shown at developmental stage D27. Figure S9. Differential gene expression. Heatmaps of differential gene expression of *PdNAC* in almond fruit is shown at developmental stage D32. Figure S10. Differential gene expression. Heatmaps of differential gene expression of *PdNAC* in almond fruit is shown at developmental stage D37. Heatmaps are based on Z-score. Table S1. Identification of *PdNAC* genes. List of the *PdNAC* genes along with their gene and protein accession number is shown. Molecular weights and isoelectric points of the proteins were predicted as well. Table S2. One to one orthologous relationship of *PdNAC* with compared organisms. One to one orthologous relationship of *PdNAC* with *A. thaliana*, *P. persica*, *J. regia*, and *M. domestica* are shown. Table S3. Gene ontology analysis. Gene ontology of the *PdNAC* is given in the table showing BLAST results and GO IDs. Table S4. Z-score of *PdNAC*. Z-score of differentially expressed *PdNAC* is shown in the table. Table S5. Common significant *PdNAC* at each sampling stage. Significant *PdNAC* for each stage are given. Table S6. SRA accessions of almond RNA-seq data. SRA accession along with treatments and controls are given. Table S7. Mapped Reads Information. Unique, mismatched and duplicated repeats information is given in the table. Table S8. DESeq2 results. DESeq2 result file containing Gene ID, Log(FC), adjusted *p*-value and raw *p*-value of differentially expressed genes having significant *p*-value. Data S1. Protein sequences of *PdNAC*. Protein sequences of identified NAC of *Prunus dulcis* are given. Data S2. *Cis*-regulatory elements of *PdNAC*. *Cis*-regulatory elements of *PdNAC* along with their start and end position are provided. Data S3. DESeq2 Plot. Plots of DESeq2 are visualized depicting the normalized count and *p* values.
