# Supplementary material for: Comprehensive Analyses of NAC Transcription Factor Family in Almond (Prunus dulcis) and Their Differential Gene Expression during Fruit Development
Source: Plants (Basel). 2021 Oct 16;10(10):2200. doi: 10.3390/plants10102200 (PMC8541688; doi:10.3390/plants10102200)
Supplement: Supplementary file 1 [file plants-10-02200-s001.zip › Supplementary Files/Supplementary Data S3.pdf]

# DESeq2: Plots for Visualization of DESeq2 Results Analysis

| Day of Sampling After Flowering | Normal/Abnormal Replicate No. | SRR Accession | Sample No. (DESeq2) |
|---|---|---|---|
| D12 | N1 | SRR10189198 | 25 |
| | N2 | SRR10189199 | 26 |
| | N3 | SRR10189200 | 27 |
| | A1 | SRR10189216 | 28 |
| | A2 | SRR10189217 | 29 |
| | A3 | SRR10189218 | 30 |
| D17 | N1 | SRR10189201 | 19 |
| | N2 | SRR10189202 | 20 |
| | N3 | SRR10189203 | 21 |
| | A1 | SRR10189219 | 22 |
| | A2 | SRR10189220 | 23 |
| | A3 | SRR10189221 | 24 |
| D22 | N1 | SRR10189204 | 13 |
| | N2 | SRR10189205 | 14 |
| | N3 | SRR10189206 | 15 |
| | A1 | SRR10189222 | 16 |
| | A2 | SRR10189223 | 17 |
| | A3 | SRR10189224 | 18 |
| D27 | N1 | SRR10189207 | 7 |
| | N2 | SRR10189208 | 8 |
| | N3 | SRR10189209 | 9 |
| | A1 | SRR10189225 | 10 |
| | A2 | SRR10189226 | 11 |
| | A3 | SRR10189227 | 12 |
| D32 | N1 | SRR10189210 | 1 |
| | N2 | SRR10189211 | 2 |
| | N3 | SRR10189212 | 3 |
| | A1 | SRR10189228 | 4 |
| | A2 | SRR10189229 | 5 |
| | A3 | SRR10189230 | 6 |
| D37 | N1 | SRR10189213.1 | 31 |
| | N3 | SRR10189214.1 | 32 |
| | N2 | SRR10189215.1 | 33 |
| | A1 | SRR10189231.1 | 34 |
| | A2 | SRR10189232.1 | 35 |
| | A3 | SRR10189233.1 | 36 |

# DESeq2: Day12-Analysis Plots

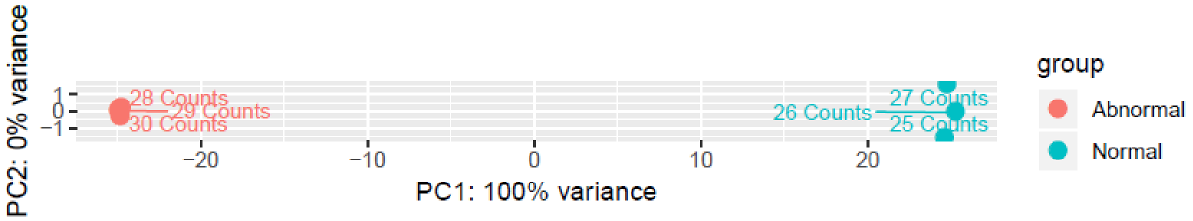


Figure 1: Figure 1: Principal component plots of the normal and abnormal samples at D12 after flowering. PC1 shows 100% variance among the normal and abnormal samples whereas PC2 shows 0% variance as all data is paired end.

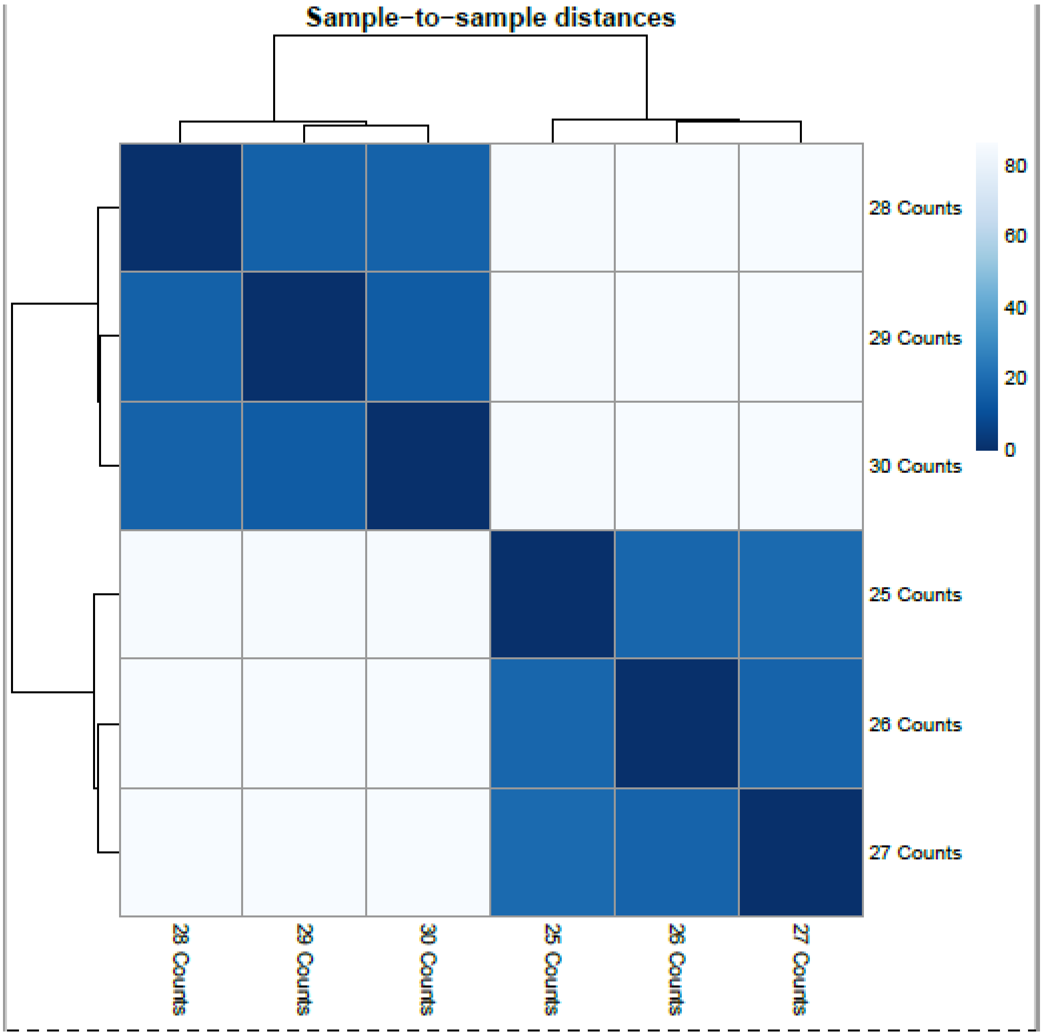


Figure 2: Heatmap of the sample-to-sample distances is based on the normalized count. These are grouped on the basis of normal and abnormal samples at D12.

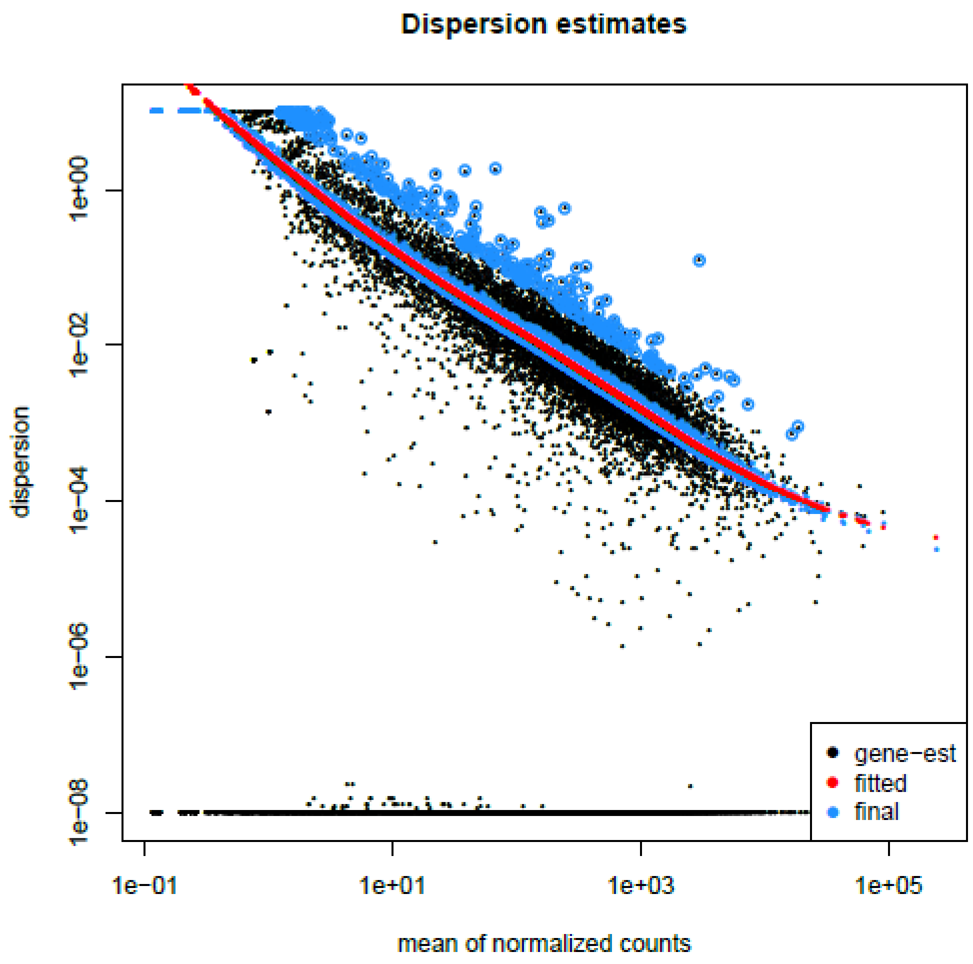


Figure 3: Dispersion estimates: gene-wise estimates (black), the fitted values (red), and the final maximum a posteriori estimates used in testing (blue) for D12 using DESeq2.

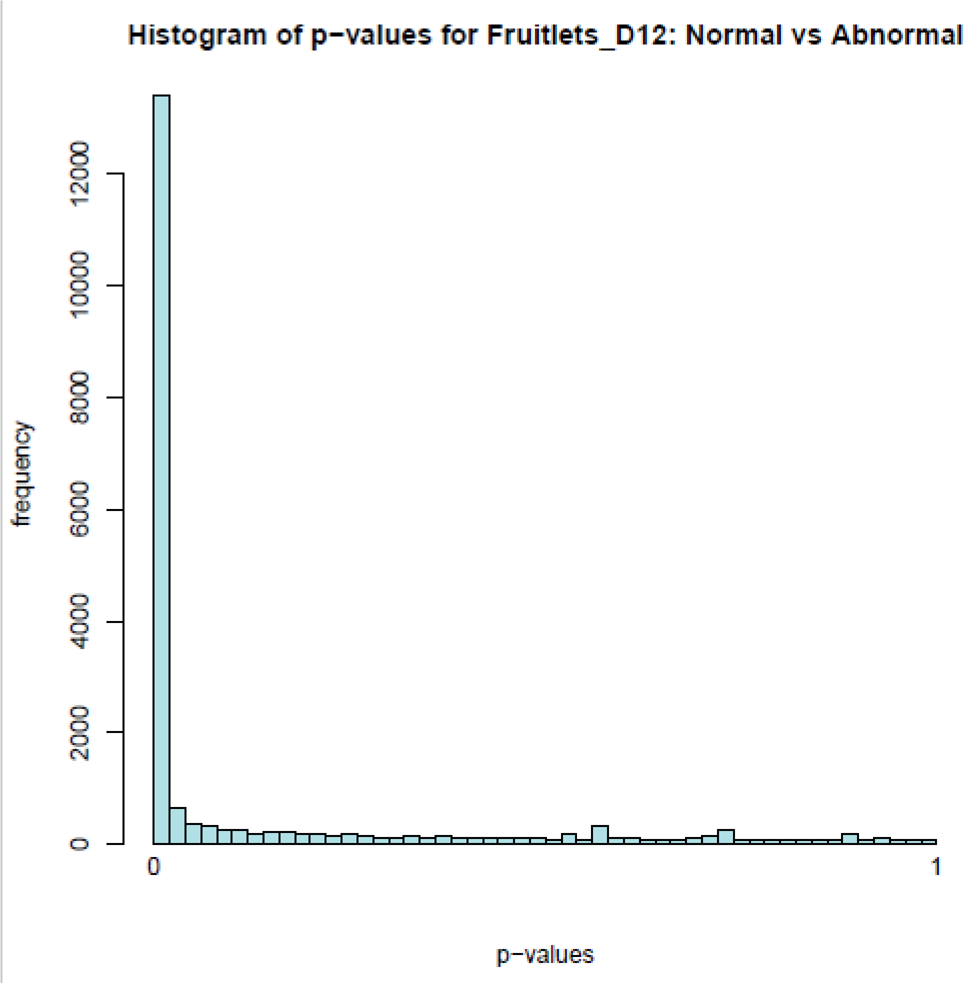


Figure 4: Histogram of p-values for the genes in the comparison between the 2 levels (normal and abnormal) of the 1st factor (normal vs abnormal fruitlets samples) at D12. X-axis has p-values whereas Y-axis represents the frequencies of genes.

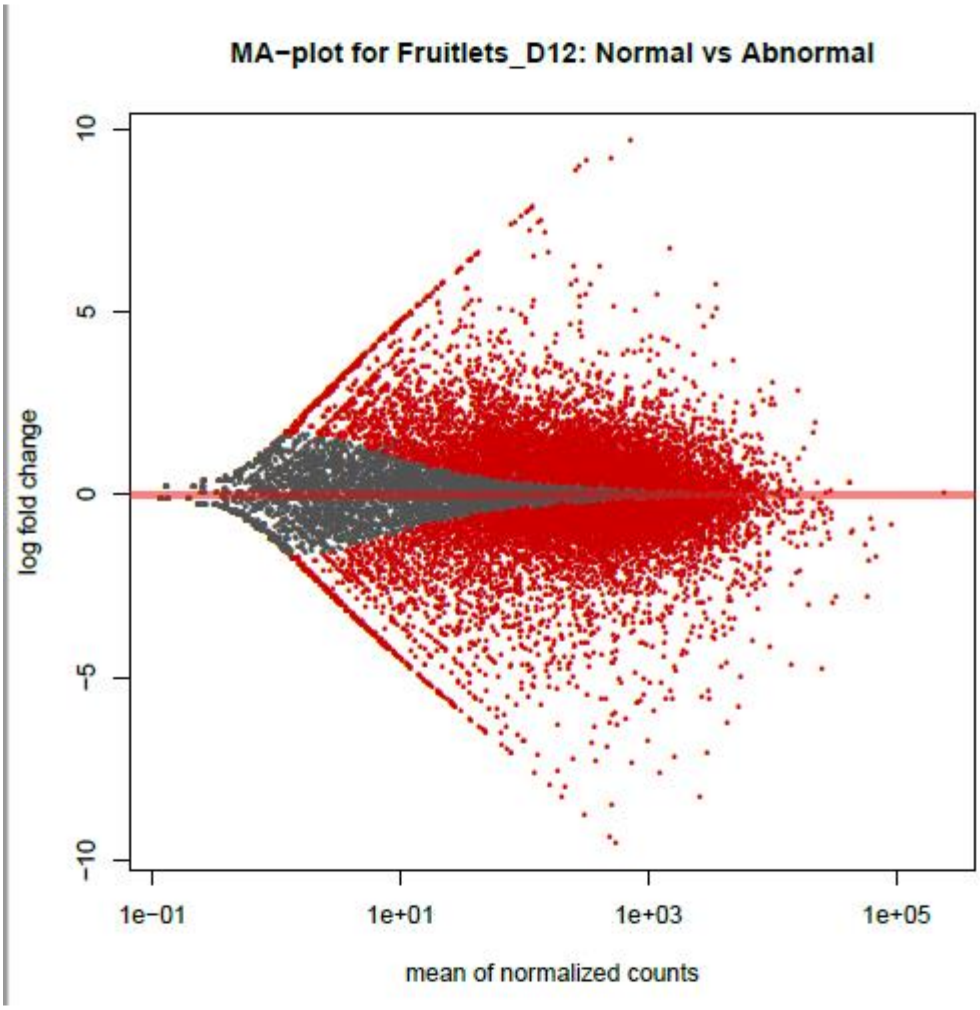


Figure 5: MA plot displays the global view of the relationship between the expression change of conditions (log ratios, M), the average expression strength of the genes (average mean, A), and the ability of the algorithm to detect differential gene expression. The genes that passed the significance threshold (adjusted p-value < 0.1) are coloured in red. X-axis shows represents mean of normalized counts whereas Y-axis shows log fold change. Log2 fold changes are based on normal vs abnormal samples.

# DESeq2: Day17-Analysis Plots

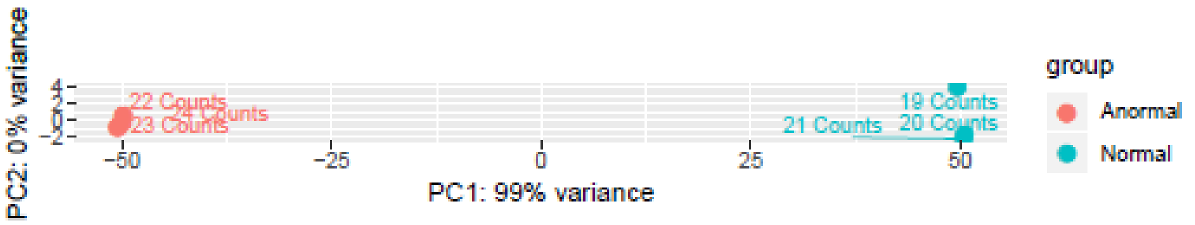


Figure 6: Figure 1: Principal component plots of the normal and abnormal samples at D17 after flowering. PC1 shows 100% variance among the normal and abnormal samples whereas PC2 shows 0% variance as all data is paired end.

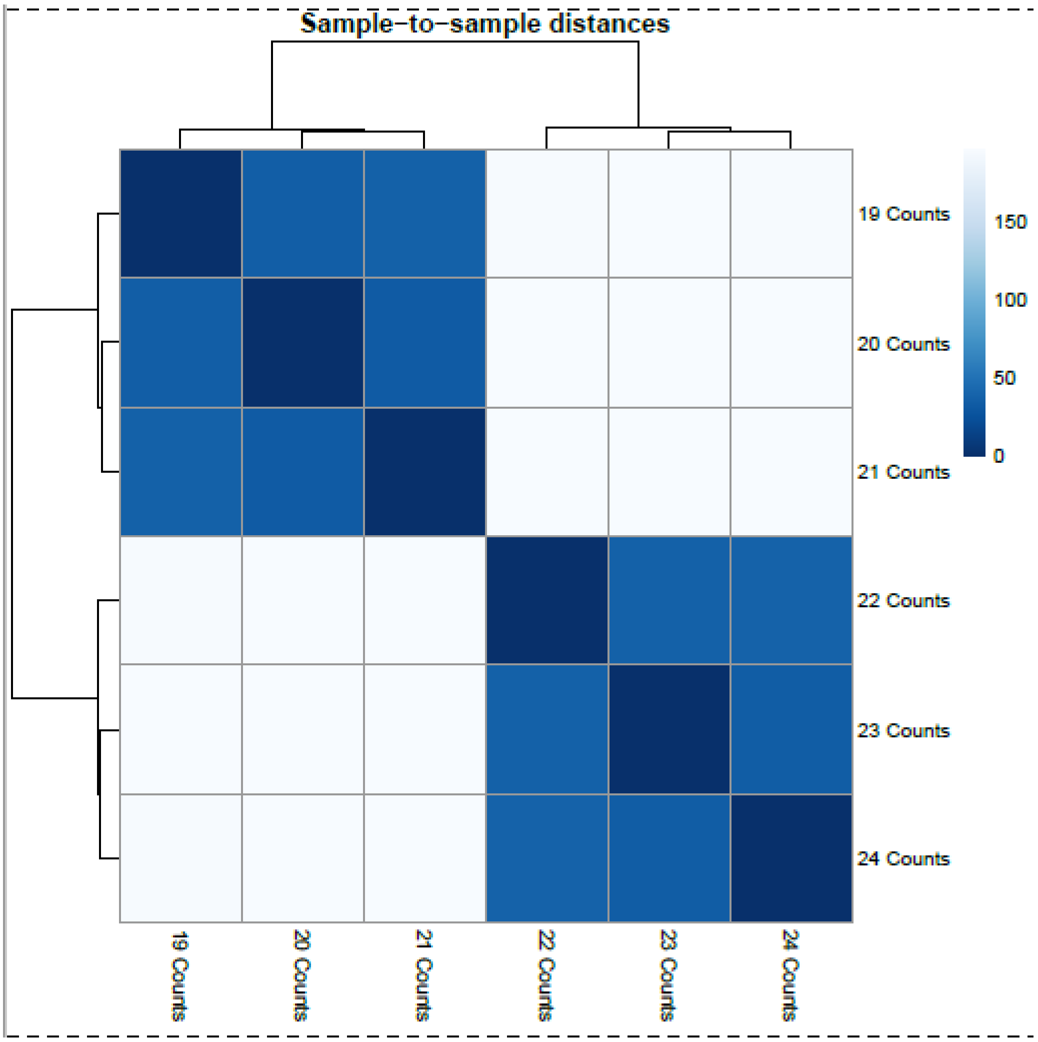


Figure 7: Heatmap of the sample-to-sample distances is based on the normalized count. These are grouped on the basis of normal and abnormal samples at D17.

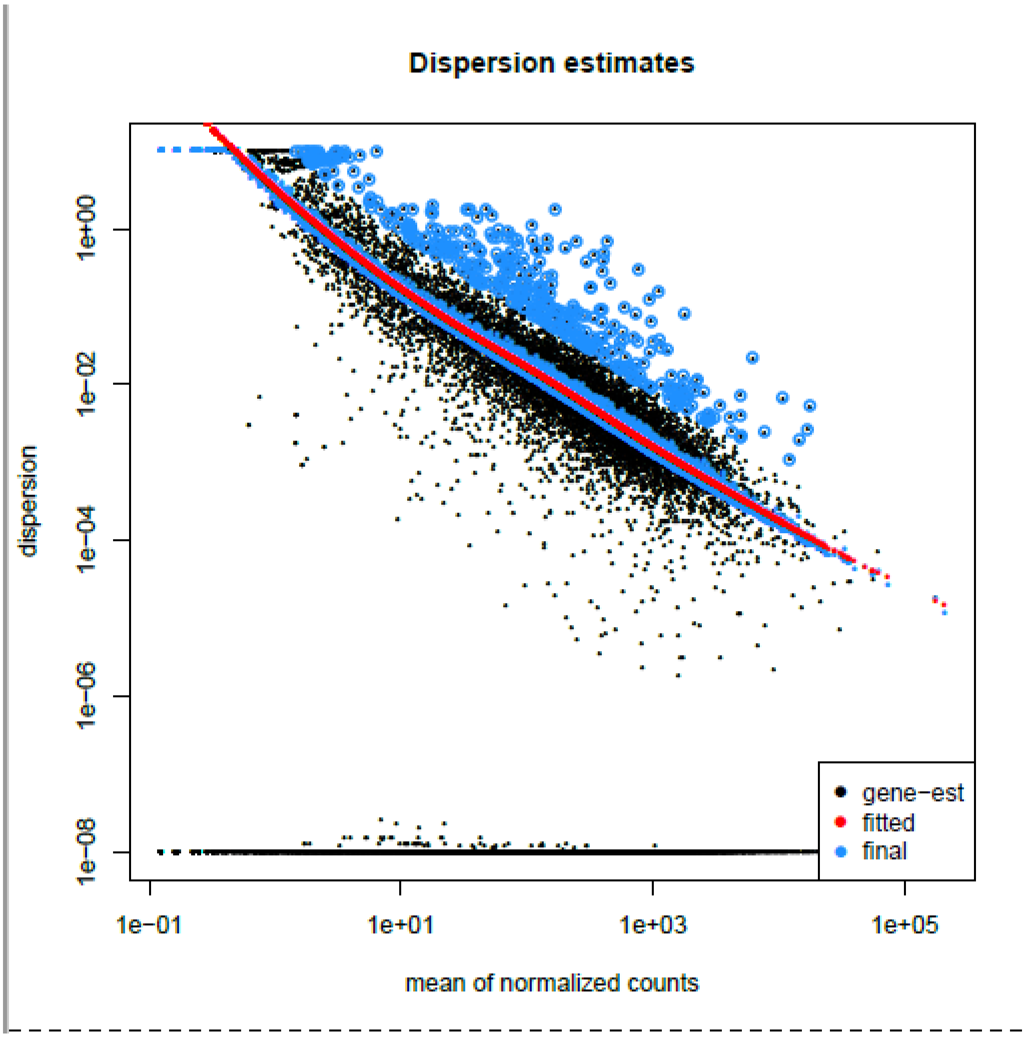


Figure 8: Dispersion estimates: gene-wise estimates (black), the fitted values (red), and the final maximum a posteriori estimates used in testing (blue) for D17 using DESeq2.

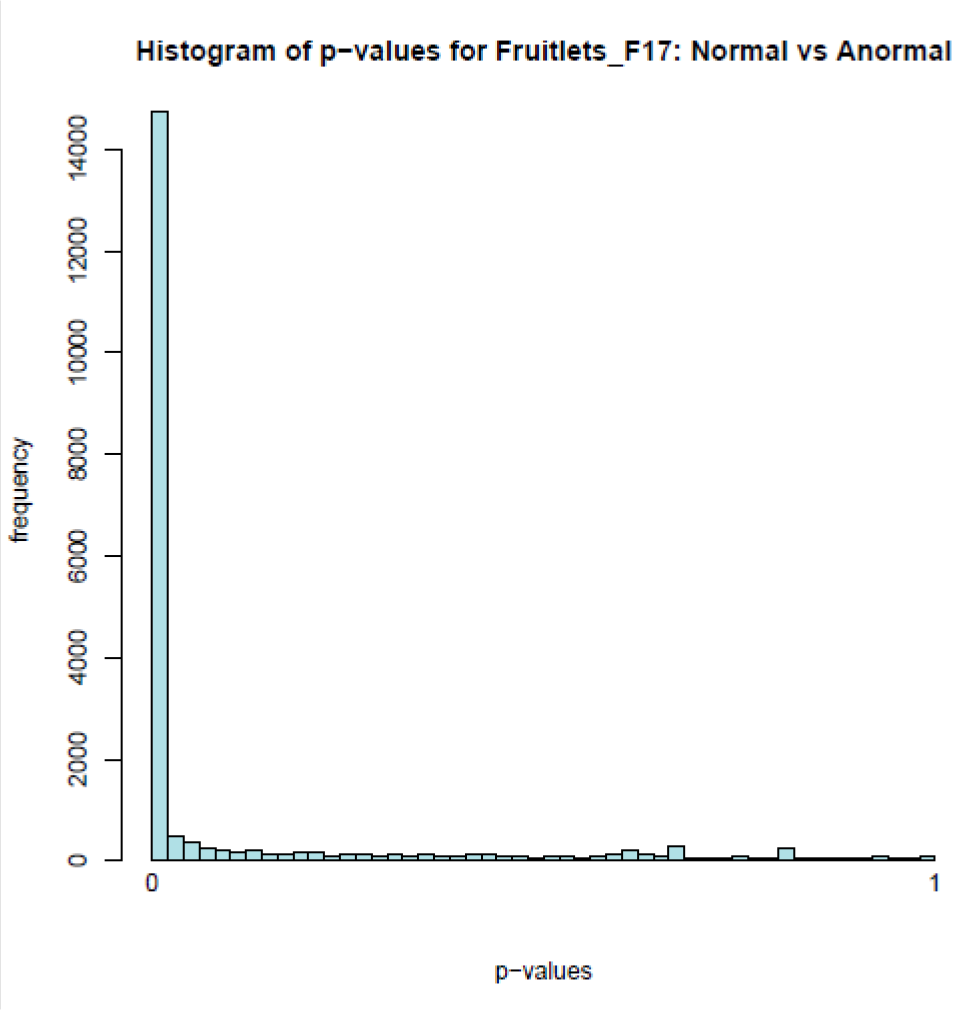


Figure 9: Histogram of p-values for the genes in the comparison between the 2 levels (normal and abnormal) of the 1st factor (normal vs abnormal fruitlets samples) at D17. It was generated by DESeq2. P-value represents the statistical significance of change in expression.

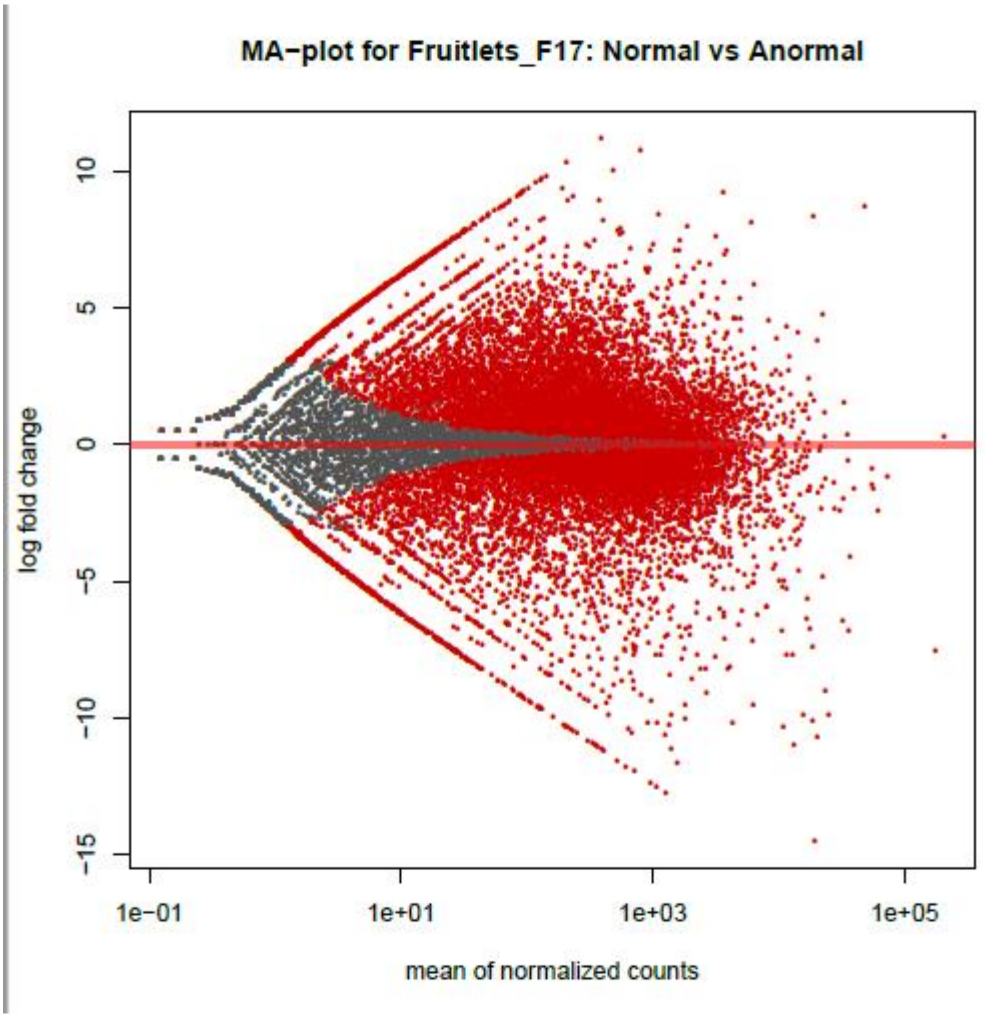


Figure 5: MA plot displays the global view of the relationship between the expression change of conditions (log ratios, M), the average expression strength of the genes (average mean, A), and the ability of the algorithm to detect differential gene expression. The genes that passed the significance threshold (adjusted p-value < 0.1) are coloured in red. X-axis shows represents mean of normalized counts whereas Y-axis shows log fold change. Log2 fold changes are based on normal vs abnormal samples.

# DESeq2: Day22-Analysis Plots

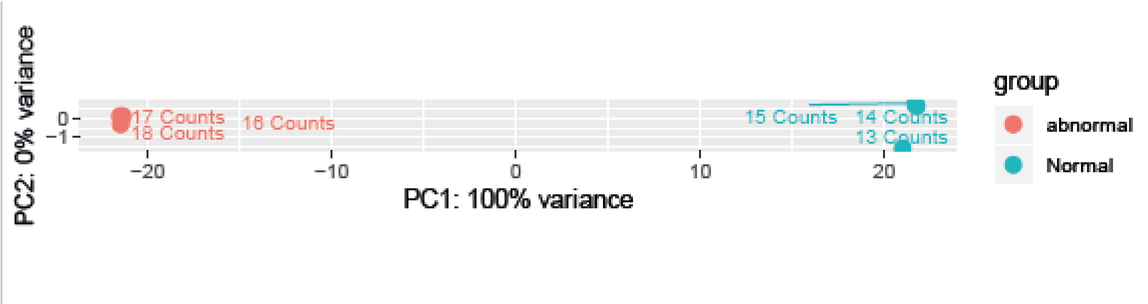


Figure 11: Figure 1: Principal component plots of the normal and abnormal samples at D22 after flowering. PC1 shows 100% variance among the normal and abnormal samples whereas PC2 shows 0% variance as all data is paired end.

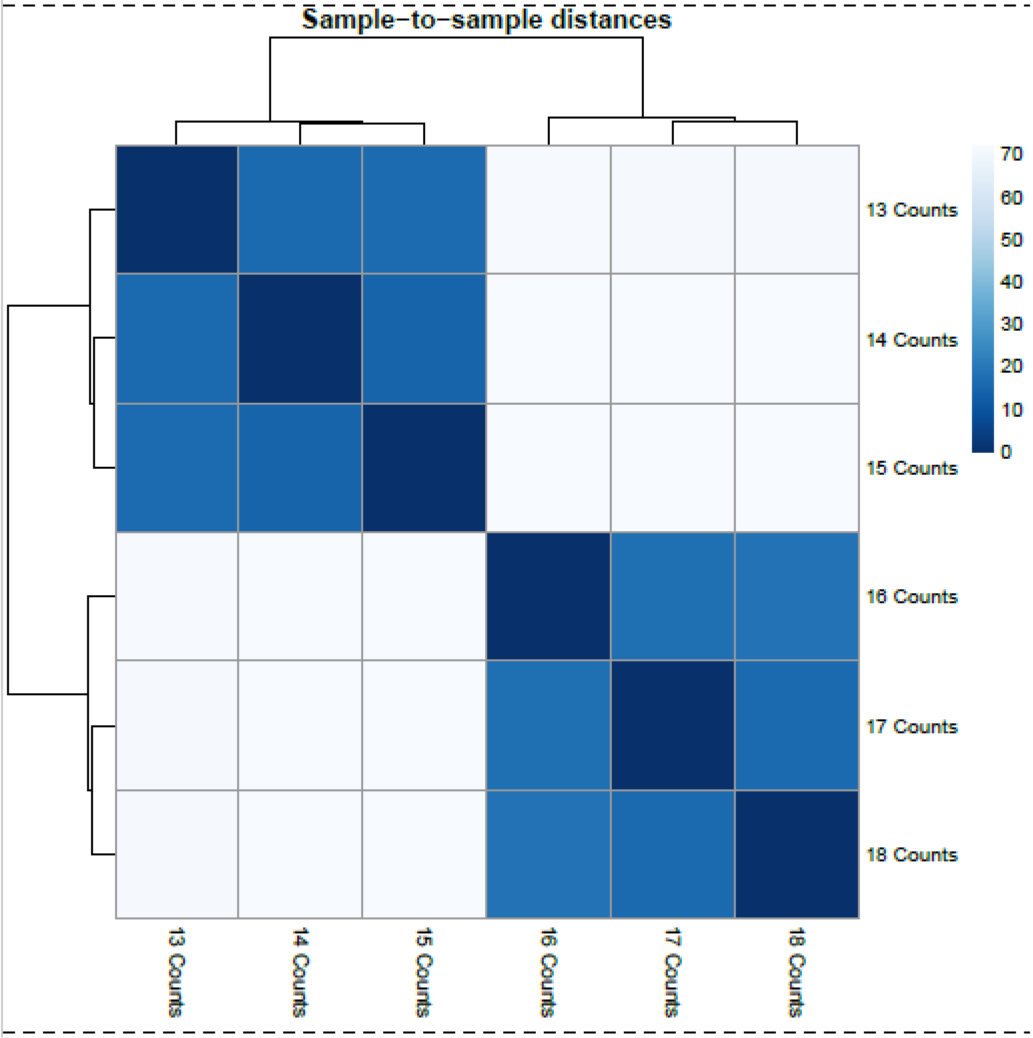


Figure 12: Heatmap of the sample-to-sample distances is based on the normalized count. These are grouped on the basis of normal and abnormal samples at D22.

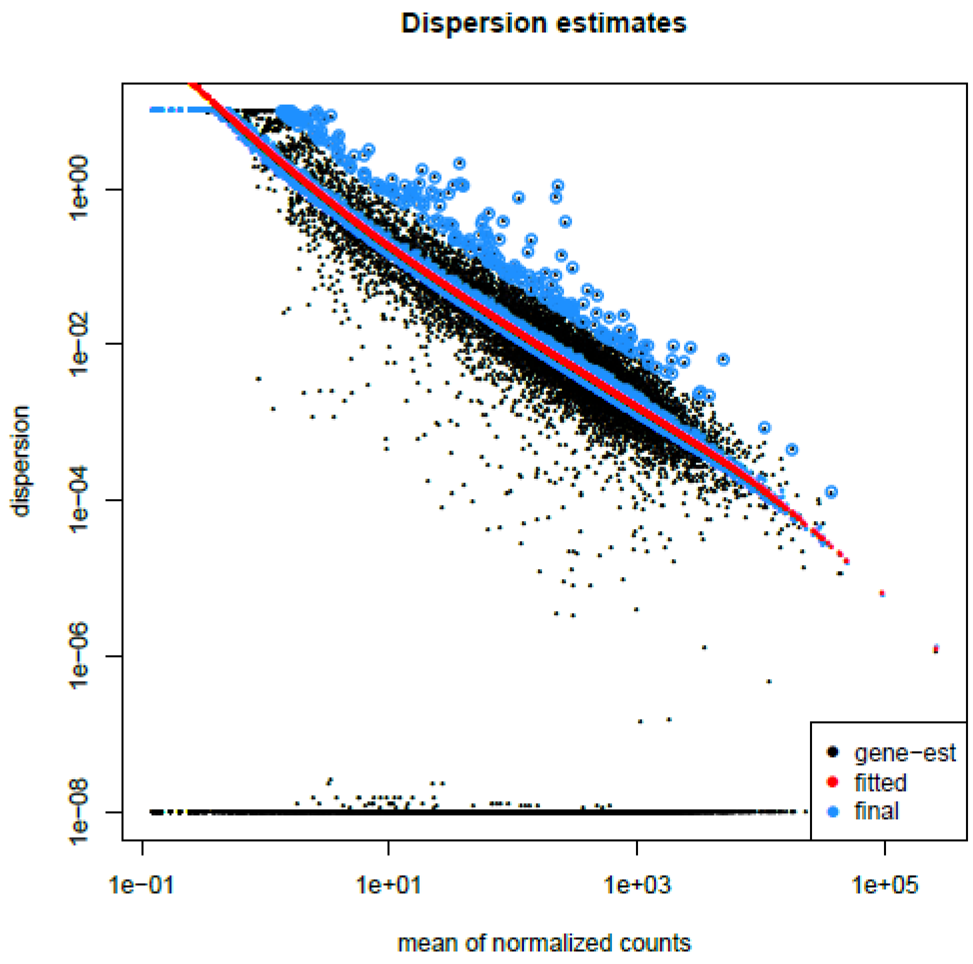


Figure 13: Dispersion estimates: gene-wise estimates (black), the fitted values (red), and the final maximum a posteriori estimates used in testing (blue) for D22 using DESeq2.

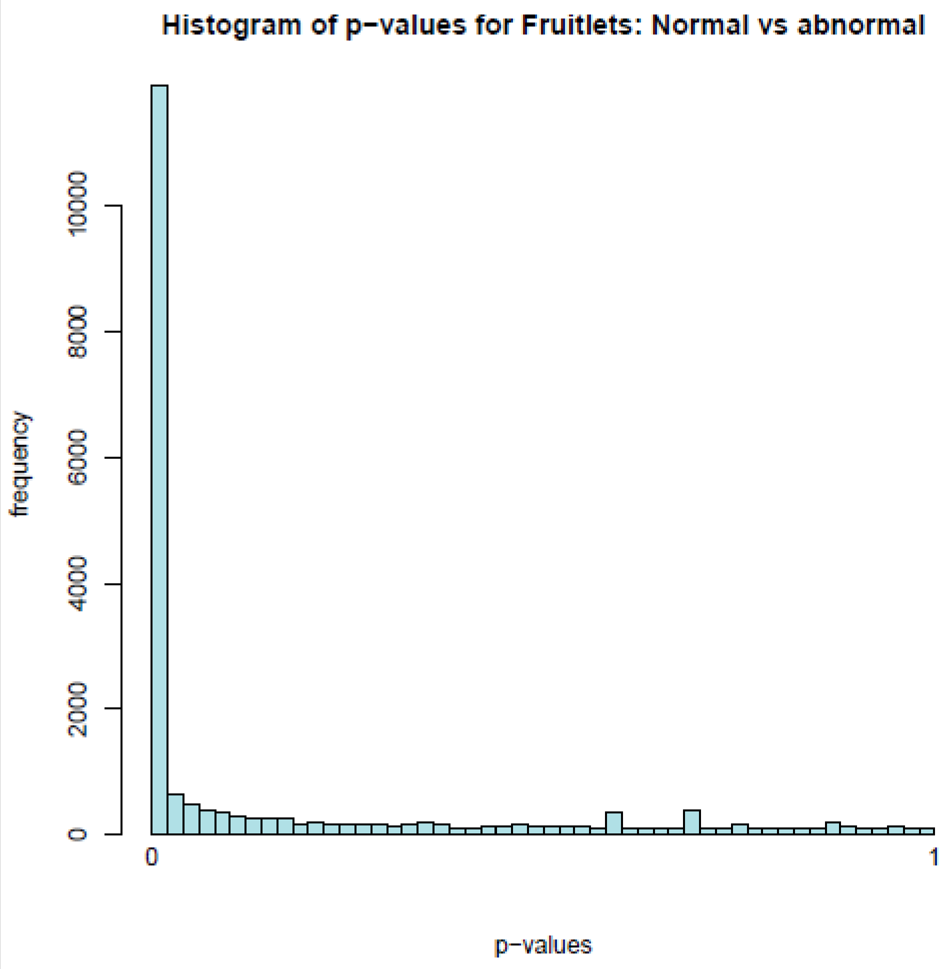


Figure 14: Histogram of p-values for the genes in the comparison between the 2 levels (normal and abnormal) of the 1st factor (normal vs abnormal fruitlets samples) at D22. It was generated by DESeq2. P-value represents the statistical significance of change in expression.

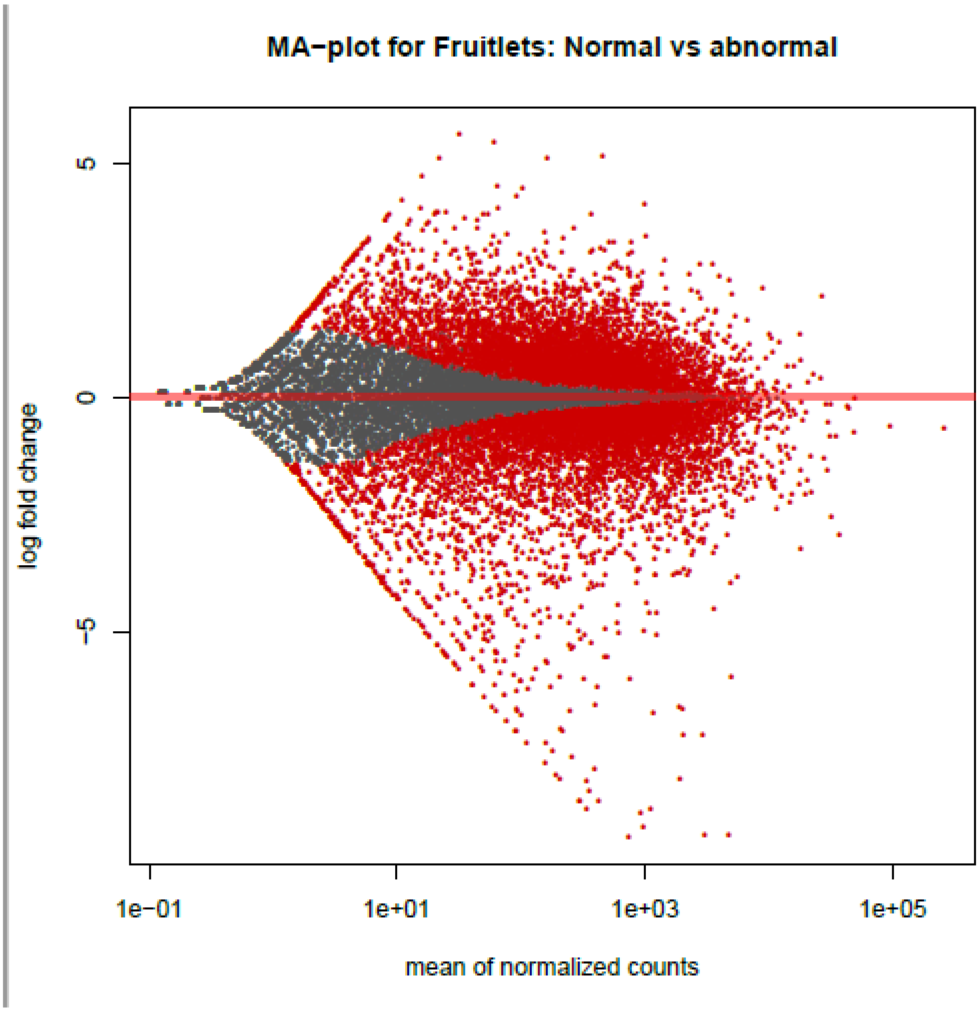


Figure 15: MA plot displays the global view of the relationship between the expression change of conditions (log ratios, M), the average expression strength of the genes (average mean, A), and the ability of the algorithm to detect differential gene expression. The genes that passed the significance threshold (adjusted p-value < 0.1) are coloured in red. X-axis shows represents mean of normalized counts whereas Y-axis shows log fold change. Log2 fold changes are based on normal vs abnormal samples.

# DESeq2: Day27-Analysis Plots

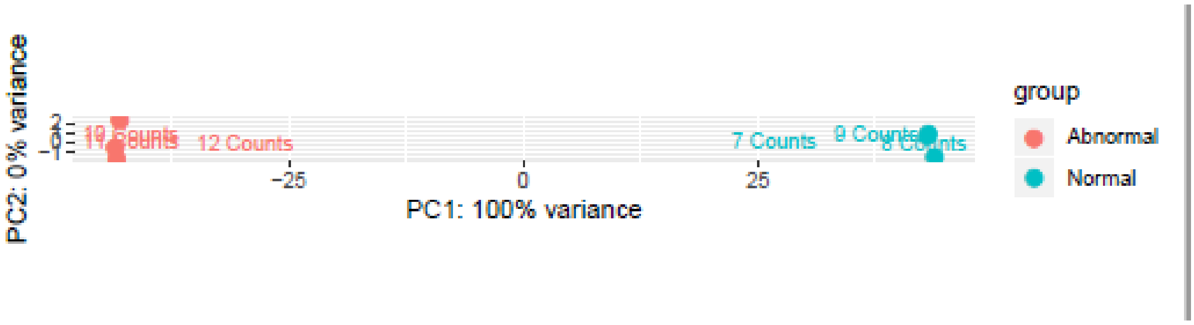


Figure 16: Figure 1: Principal component plots of the normal and abnormal samples at D27 after flowering. PC1 shows 100% variance among the normal and abnormal samples whereas PC2 shows 0% variance as all data is paired end.

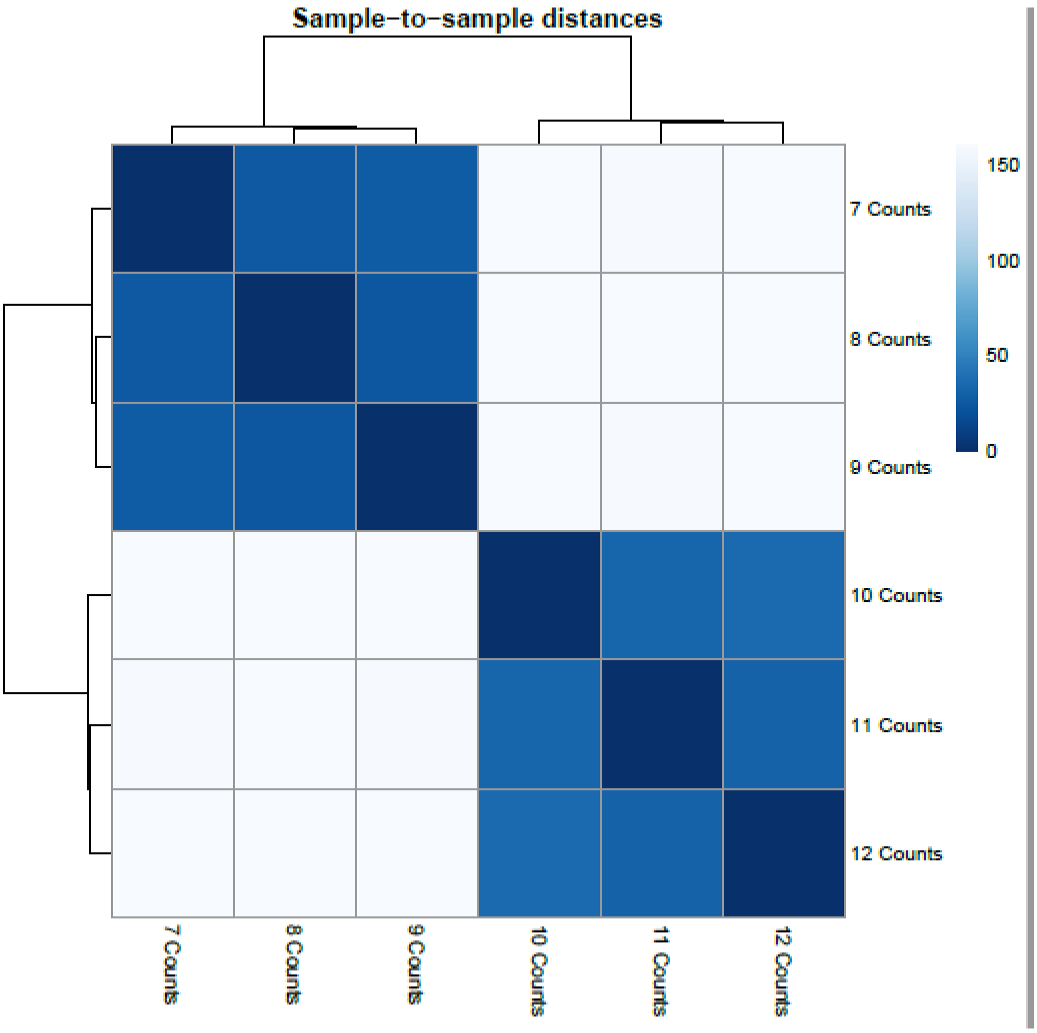


Figure 17: Heatmap of the sample-to-sample distances is based on the normalized count. These are grouped on the basis of normal and abnormal samples at D27.

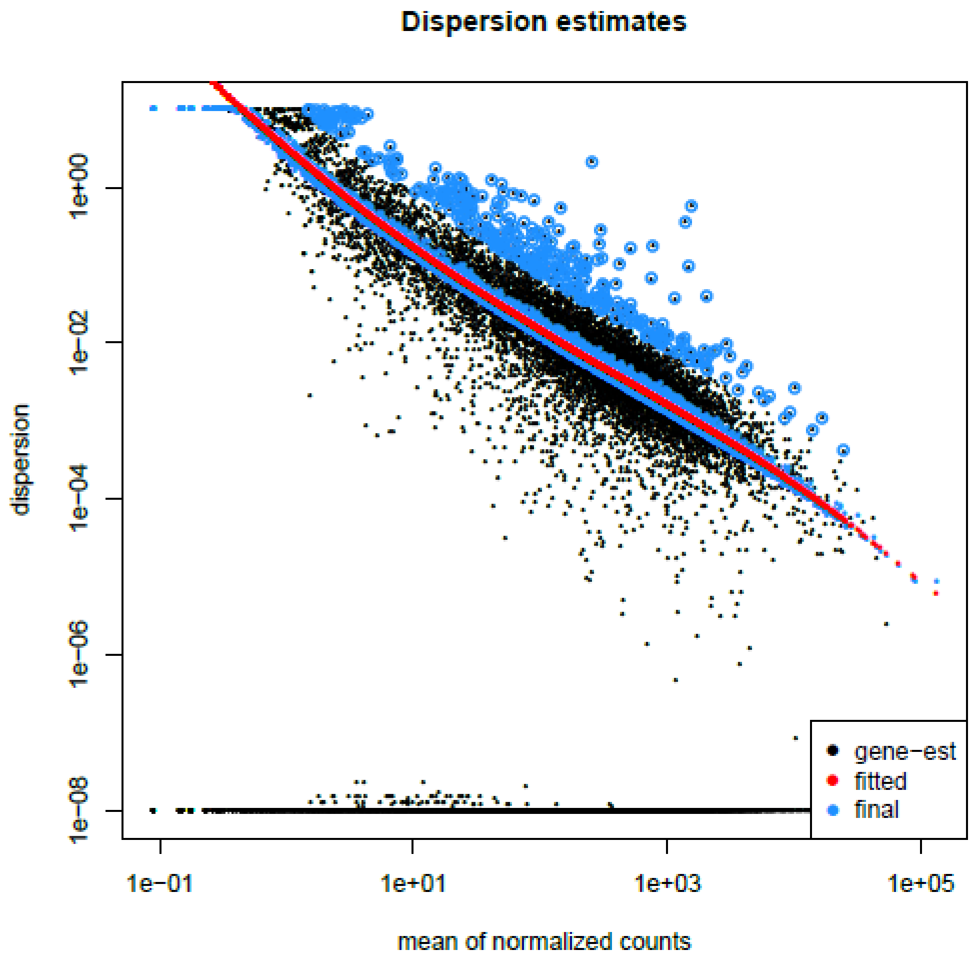


Figure 18: Dispersion estimates: gene-wise estimates (black), the fitted values (red), and the final maximum a posteriori estimates used in testing (blue) for D27 using DESeq2.

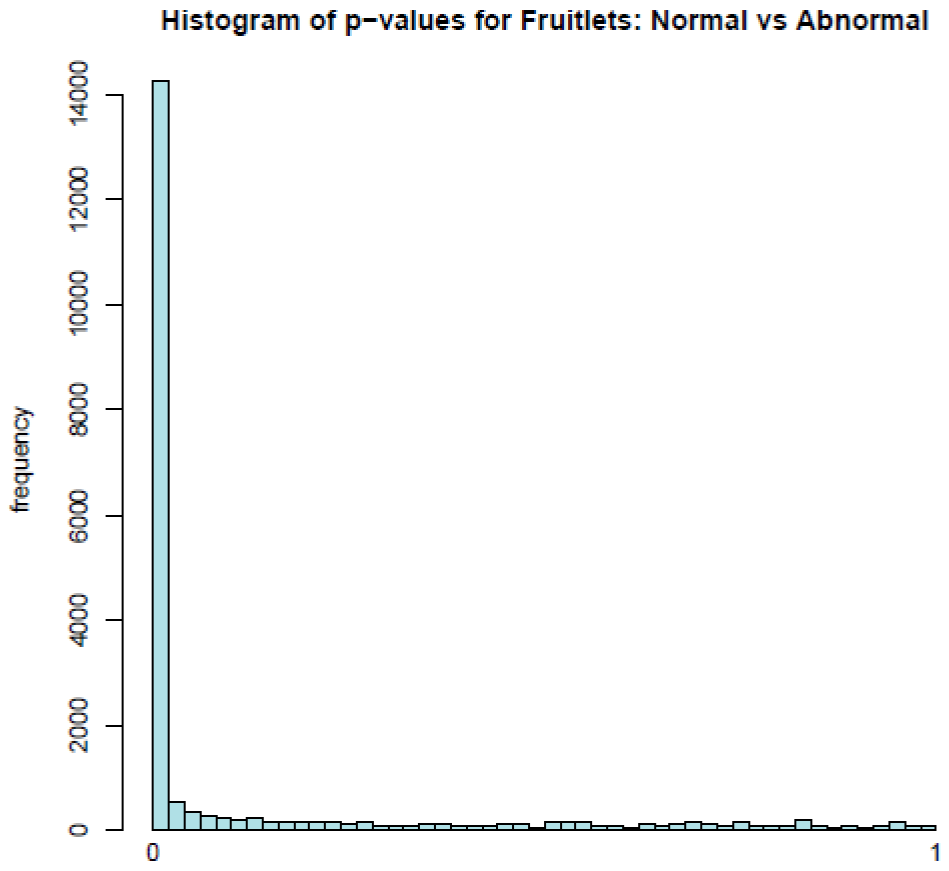


Figure 19: Histogram of p-values for the genes in the comparison between the 2 levels (normal and abnormal) of the 1st factor (normal vs abnormal fruitlets samples) at D27. It was generated by DESeq2. P-value represents the statistical significance of change in expression.

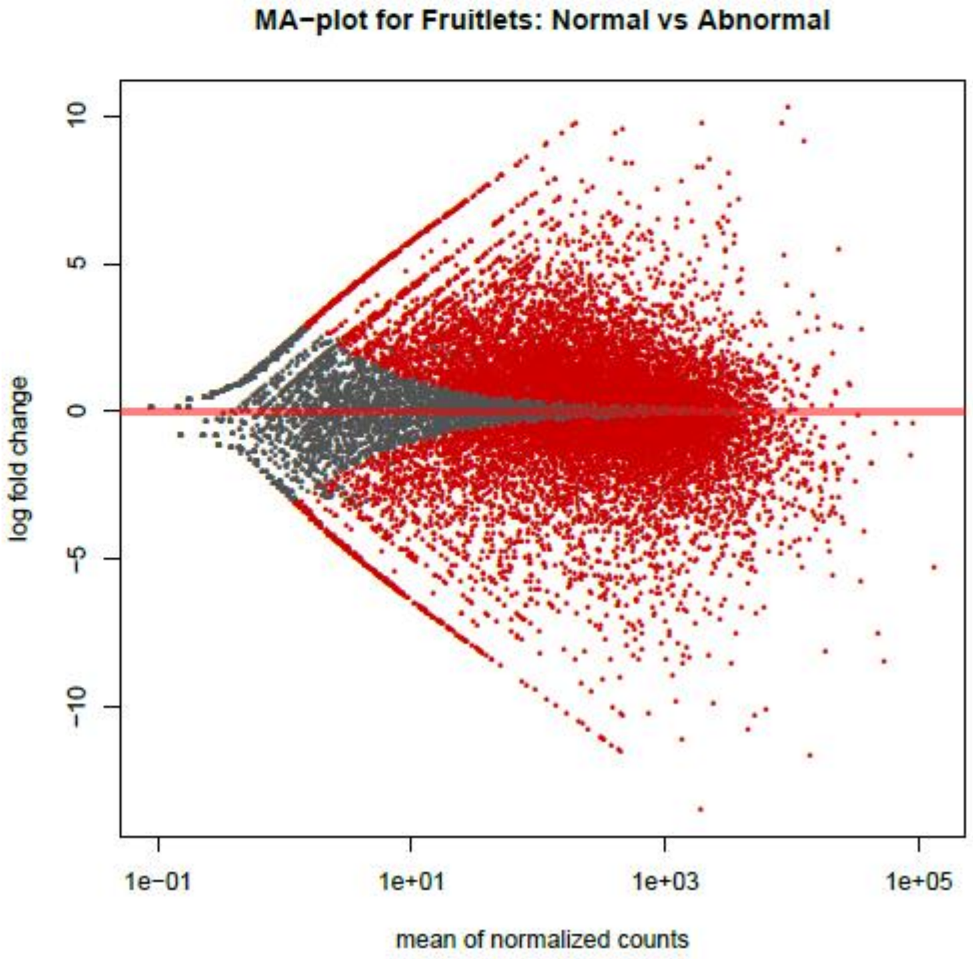


Figure 5: MA plot displays the global view of the relationship between the expression change of conditions (log ratios, M), the average expression strength of the genes (average mean, A), and the ability of the algorithm to detect differential gene expression. The genes that passed the significance threshold (adjusted p-value < 0.1) are coloured in red. X-axis shows represents mean of normalized counts whereas Y-axis shows log fold change. Log2 fold changes are based on normal vs abnormal samples.

# DESeq2: Day32-Analysis Plots

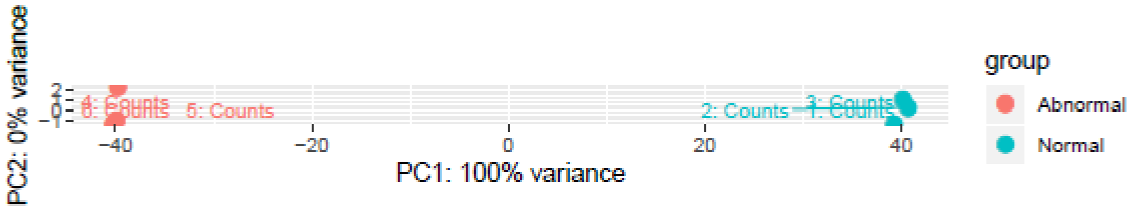


Figure 21: Figure 1: Principal component plots of the normal and abnormal samples at D32 after flowering. PC1 shows 100% variance among the normal and abnormal samples whereas PC2 shows 0% variance as all data is paired end.

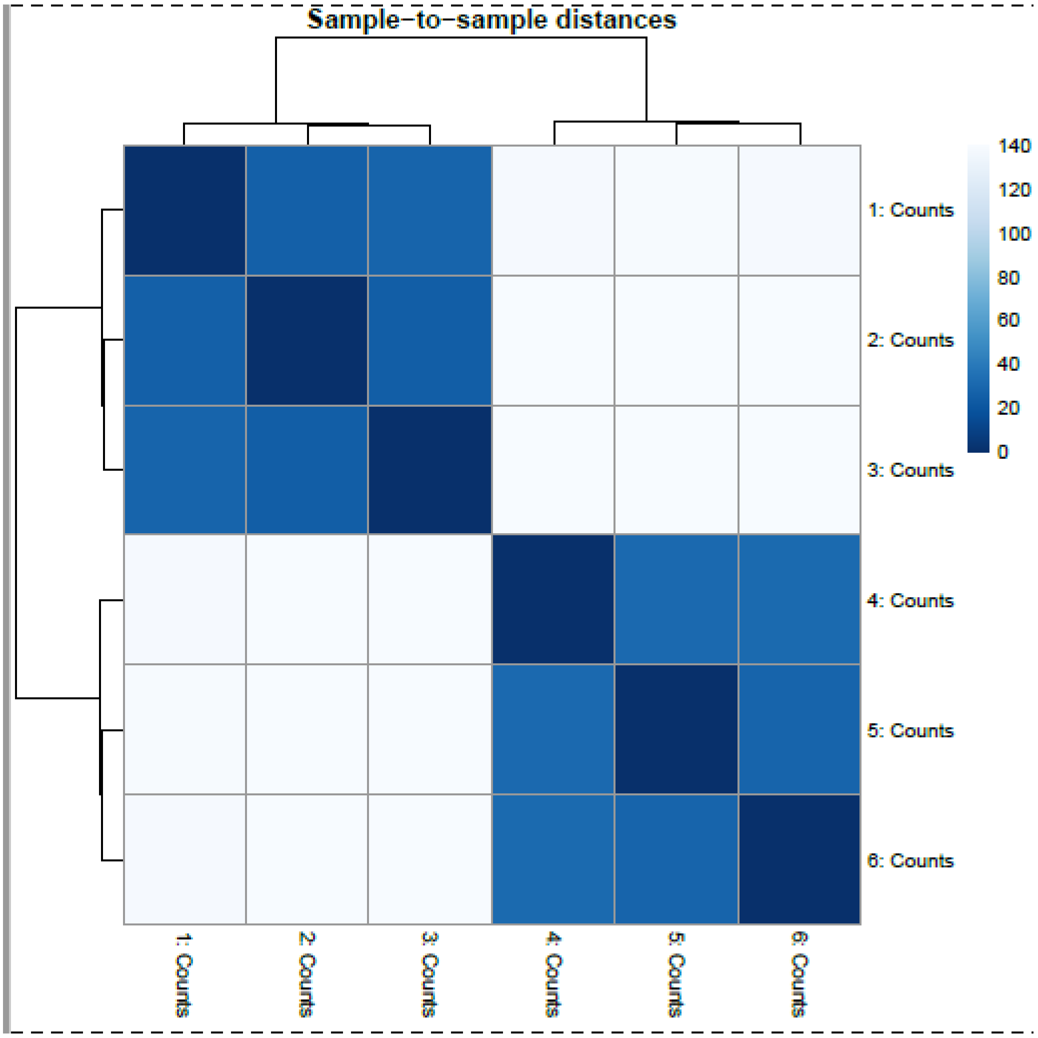


Figure 22: Heatmap of the sample-to-sample distances is based on the normalized count. These are grouped on the basis of normal and abnormal samples at D32.

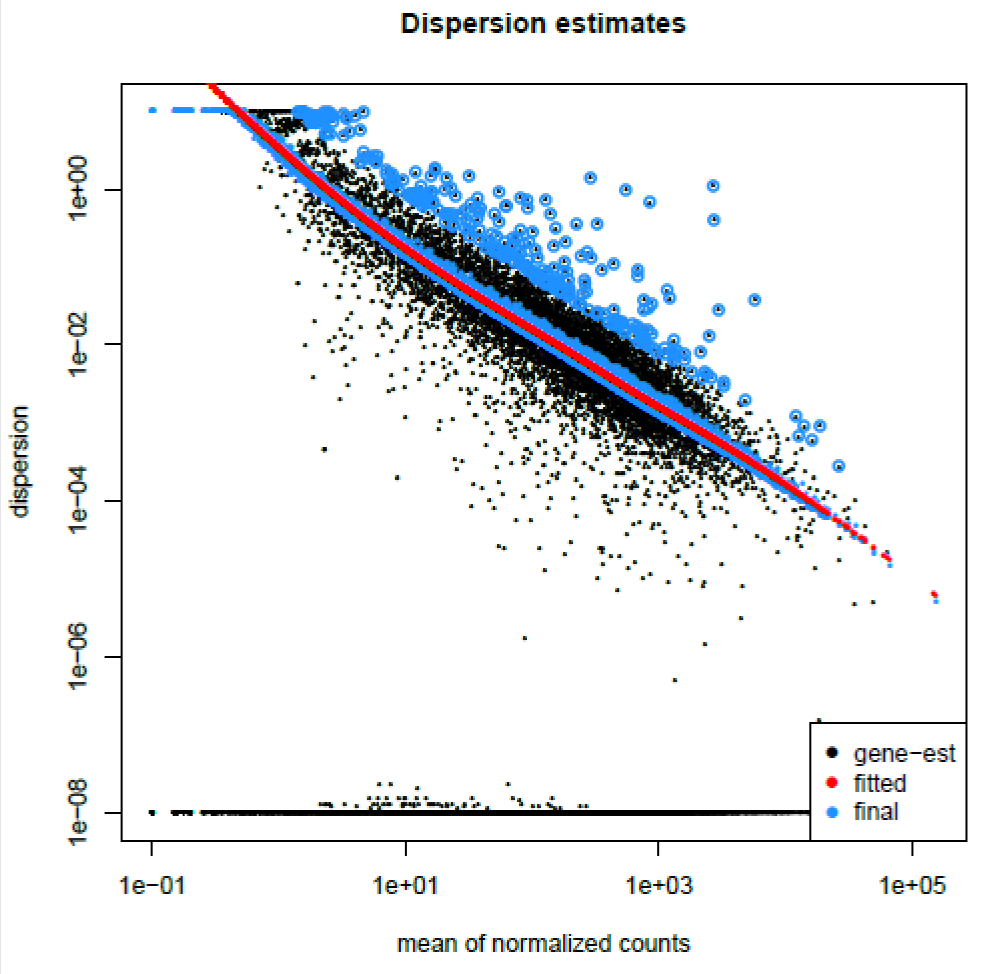


Figure 23: Dispersion estimates: gene-wise estimates (black), the fitted values (red), and the final maximum a posteriori estimates used in testing (blue) for D32 using DESeq2.

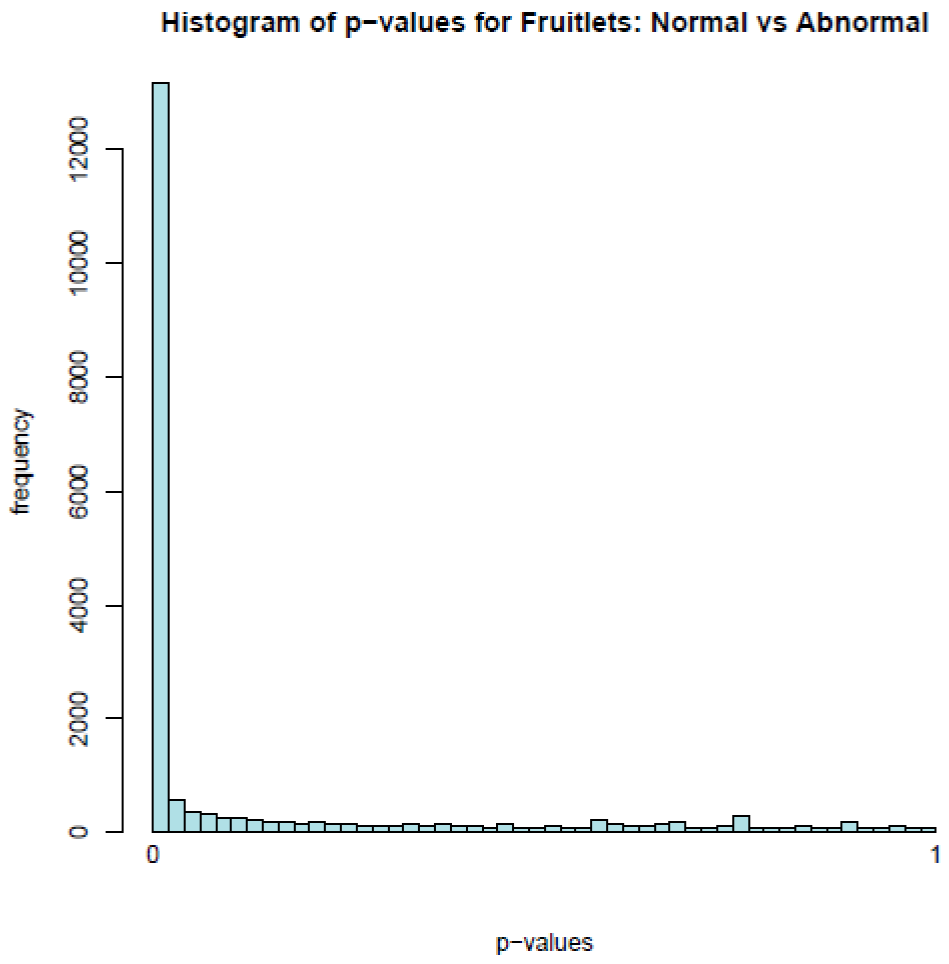


Figure 24: Histogram of p-values for the genes in the comparison between the 2 levels (normal and abnormal) of the 1st factor (normal vs abnormal fruitlets samples) at D32. It was generated by DESeq2. P-value represents the statistical significance of change in expression.

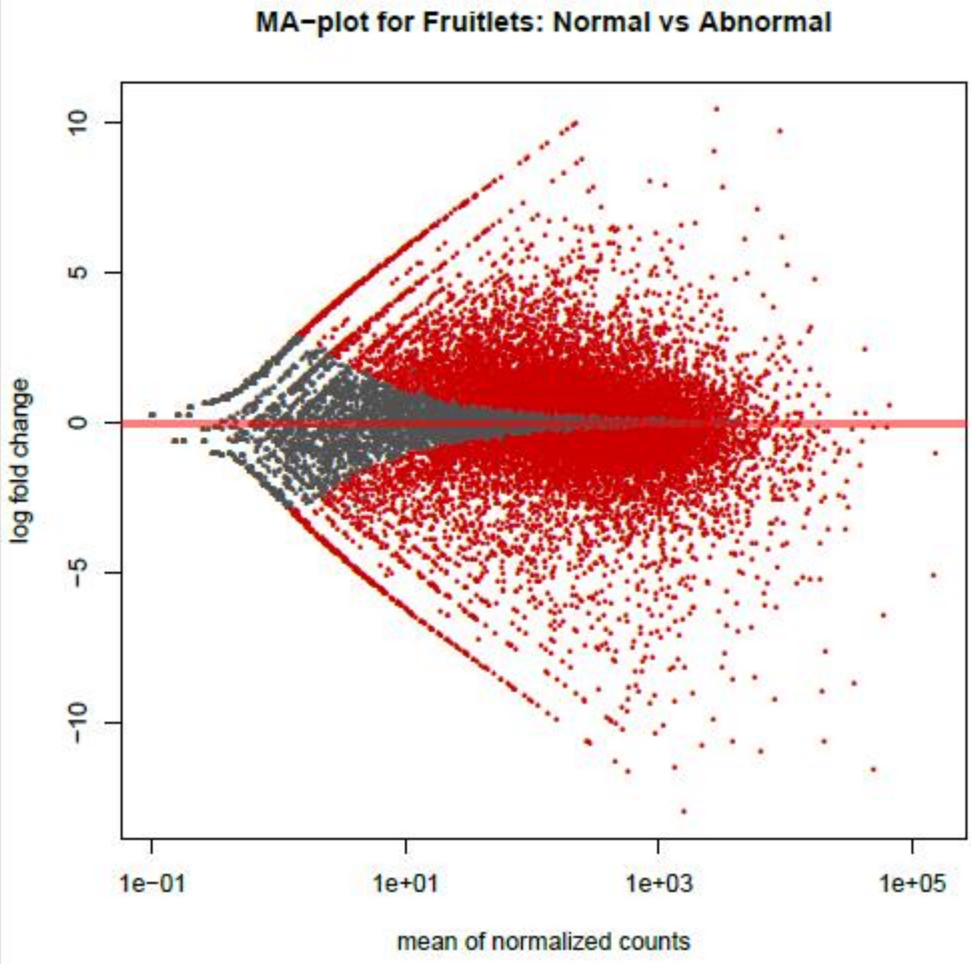


Figure 5: MA plot displays the global view of the relationship between the expression change of conditions (log ratios, M), the average expression strength of the genes (average mean, A), and the ability of the algorithm to detect differential gene expression. The genes that passed the significance threshold (adjusted p-value < 0.1) are coloured in red. X-axis shows represents mean of normalized counts whereas Y-axis shows log fold change. Log2 fold changes are based on normal vs abnormal samples.

# DESeq2: Day37-Analysis Plots

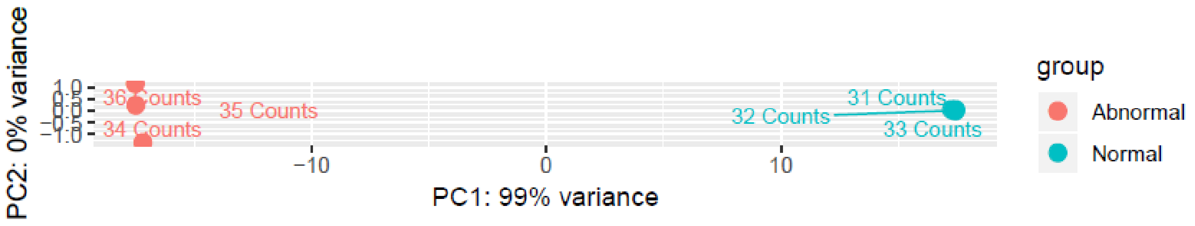


Figure 26: Figure 1: Principal component plots of the normal and abnormal samples at D37 after flowering. PC1 shows 100% variance among the normal and abnormal samples whereas PC2 shows 0% variance as all data is paired end.

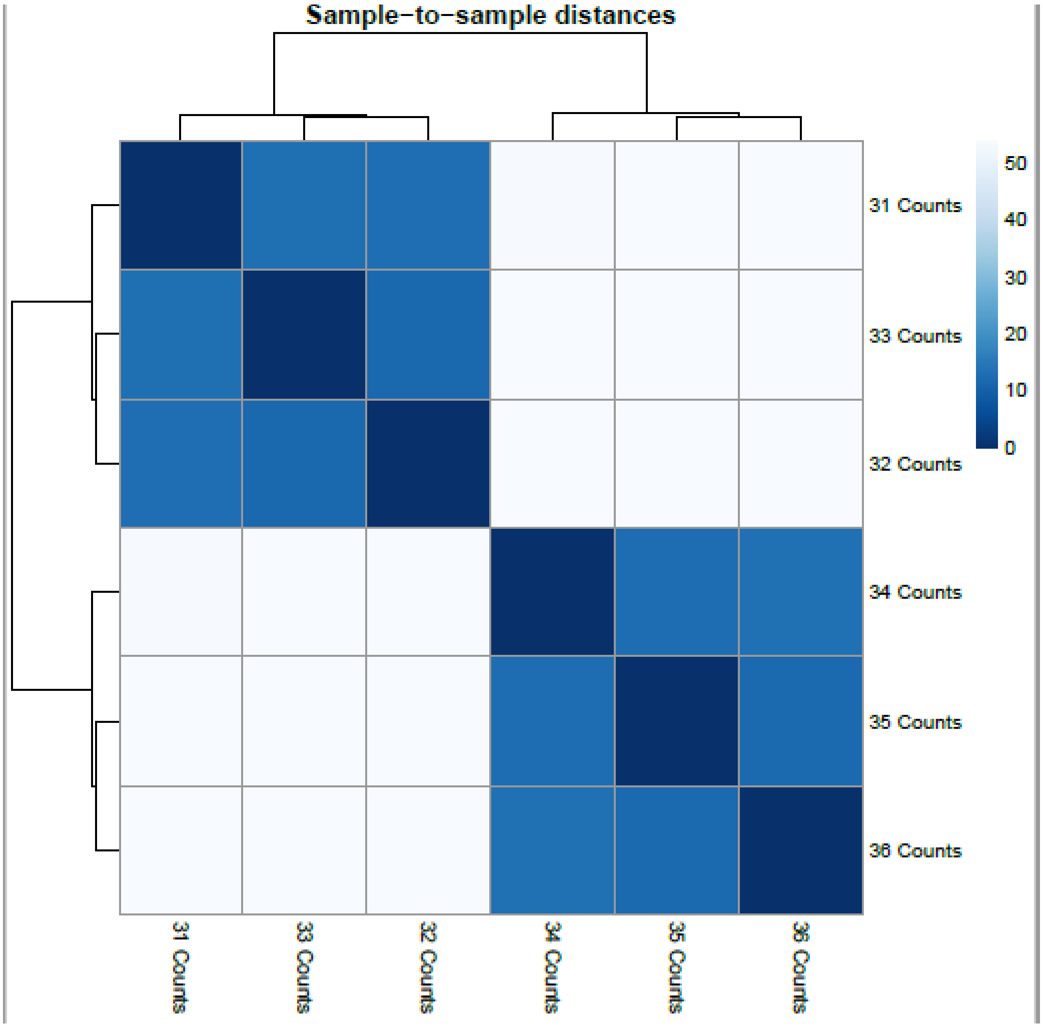


Figure 27: Heatmap of the sample-to-sample distances is based on the normalized count. These are grouped on the basis of normal and abnormal samples at D37.

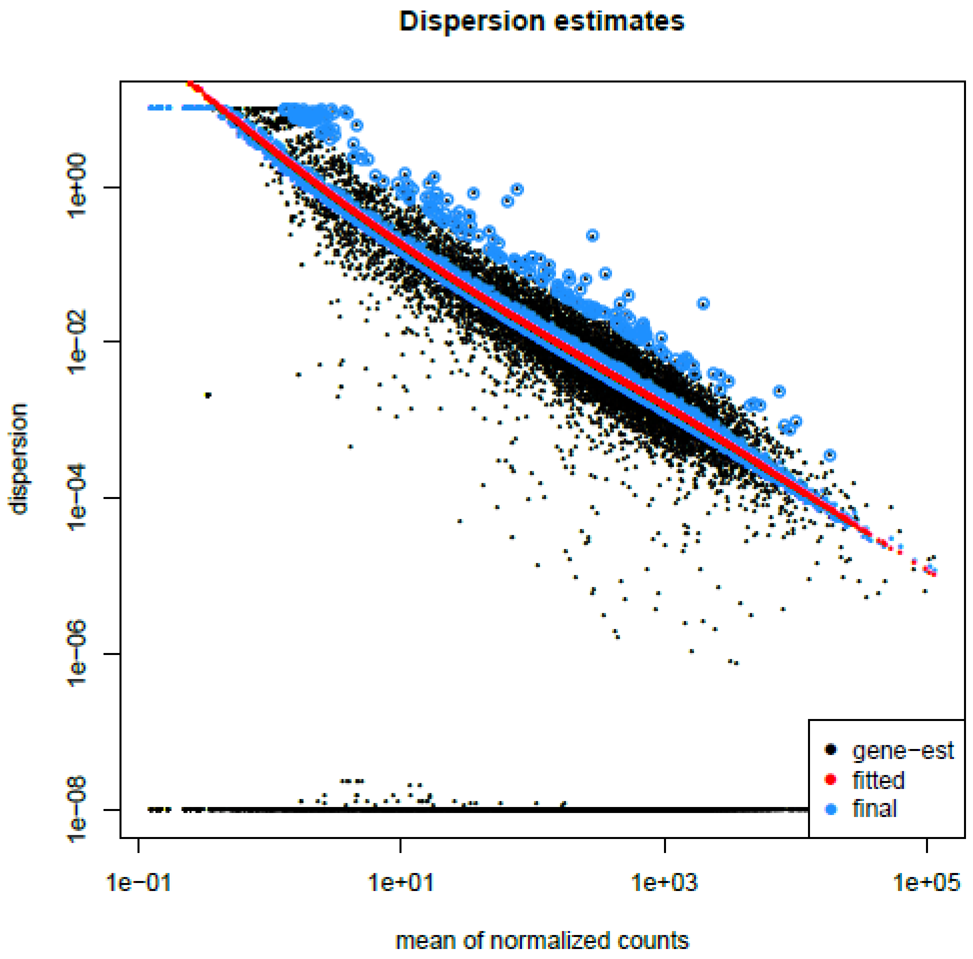


Figure 28: Dispersion estimates: gene-wise estimates (black), the fitted values (red), and the final maximum a posteriori estimates used in testing (blue) for D37 using DESeq2.

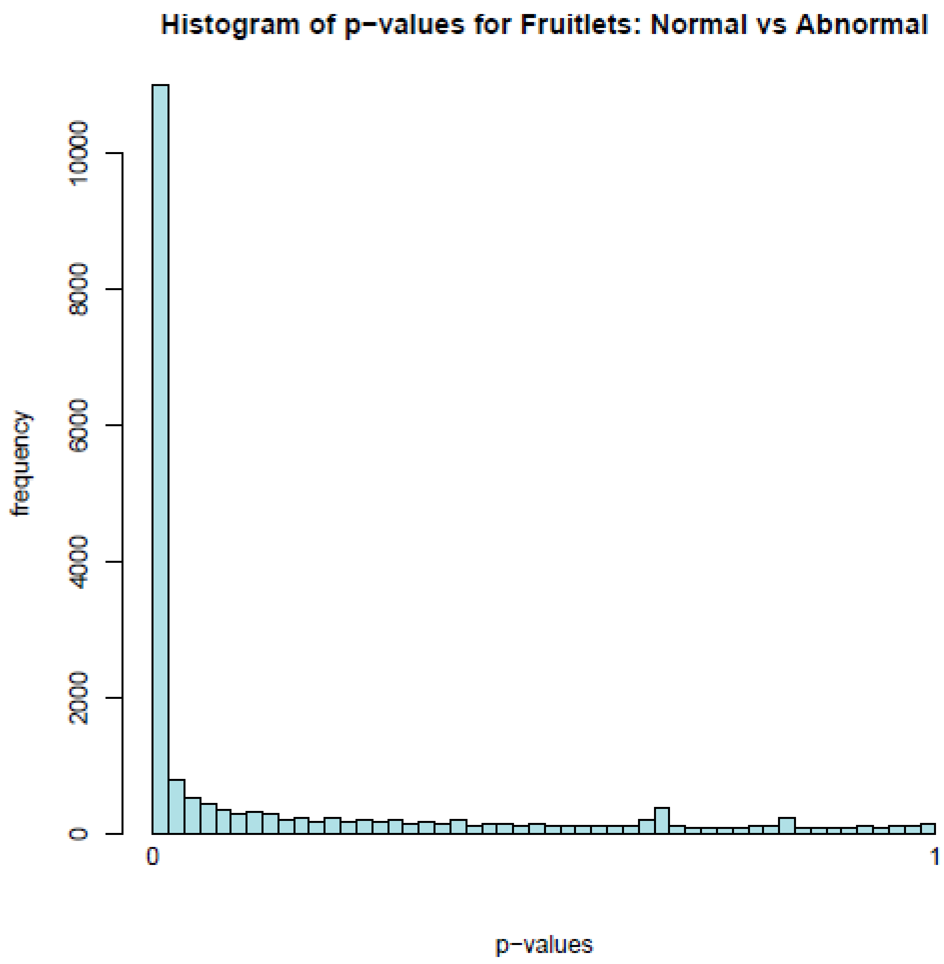


Figure 29: Histogram of p-values for the genes in the comparison between the 2 levels (normal and abnormal) of the 1st factor (normal vs abnormal fruitlets samples) at D37. It was generated by DESeq2. P-value represents the statistical significance of change in expression.

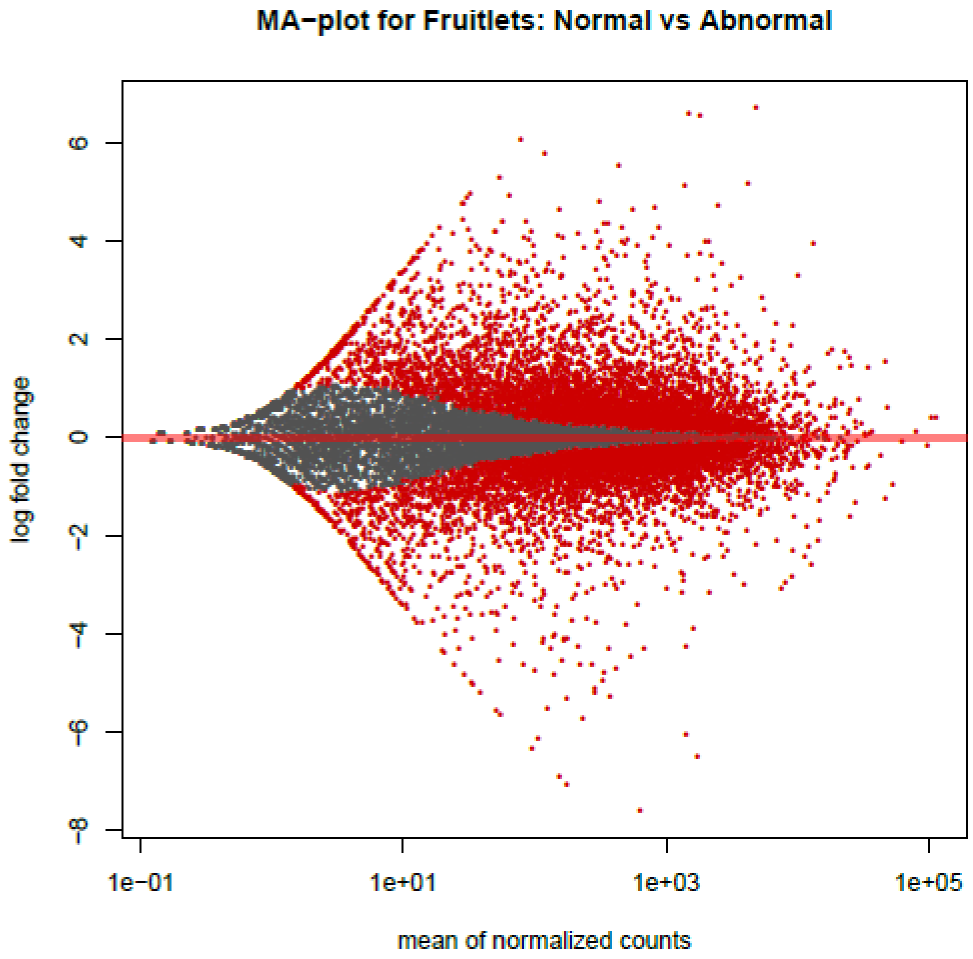


Figure 30: MA plot displays the global view of the relationship between the expression change of conditions (log ratios, M), the average expression strength of the genes (average mean, A), and the ability of the algorithm to detect differential gene expression. The genes that passed the significance threshold (adjusted p-value $< 0.1$) are coloured in red. X-axis shows represents mean of normalized counts whereas Y-axis shows log fold change. Log2 fold changes are based on normal vs abnormal samples.
