# Supplementary figures and images for: Comprehensive Analyses of NAC Transcription Factor Family in Almond (Prunus dulcis) and Their Differential Gene Expression during Fruit Development
Source: Plants (Basel). 2021 Oct 16;10(10):2200. doi: 10.3390/plants10102200 (PMC8541688; doi:10.3390/plants10102200)

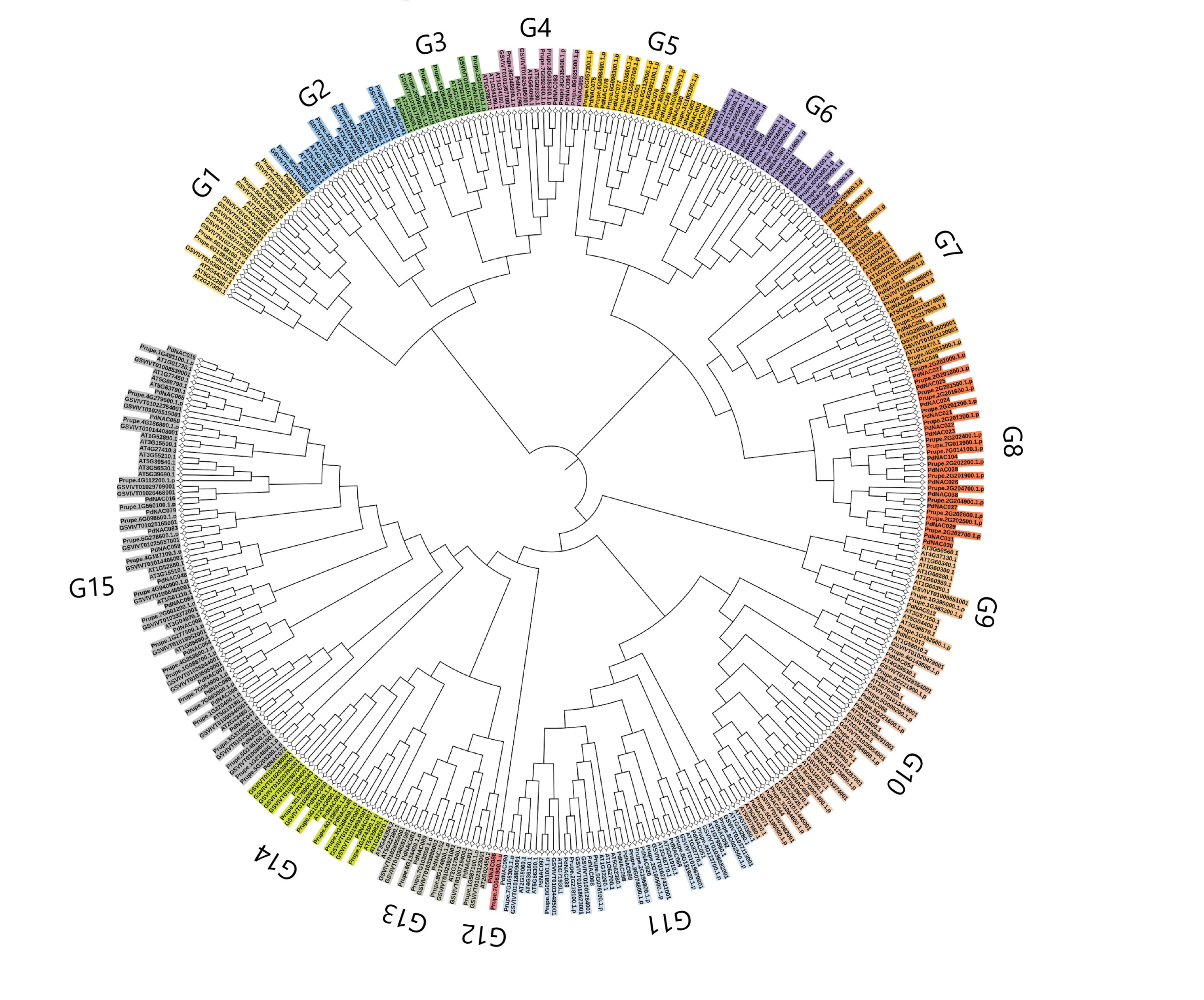

Supplement: Supplementary file 1 [file plants-10-02200-s001.zip › Supplementary Files/Supplementary Figure S1.tif]

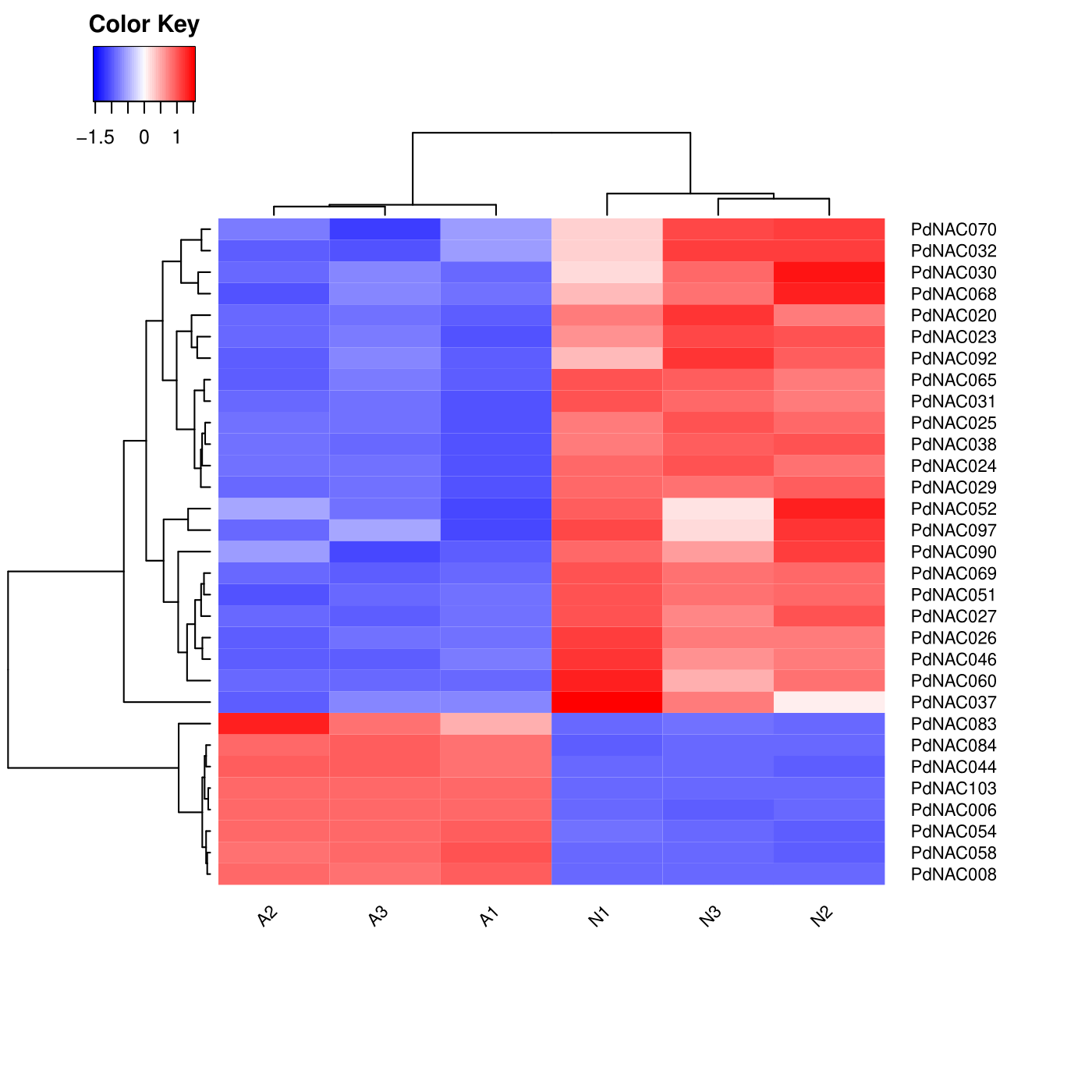

Supplement: Supplementary file 1 [file plants-10-02200-s001.zip › Supplementary Files/Supplementary Figure S10.tiff]

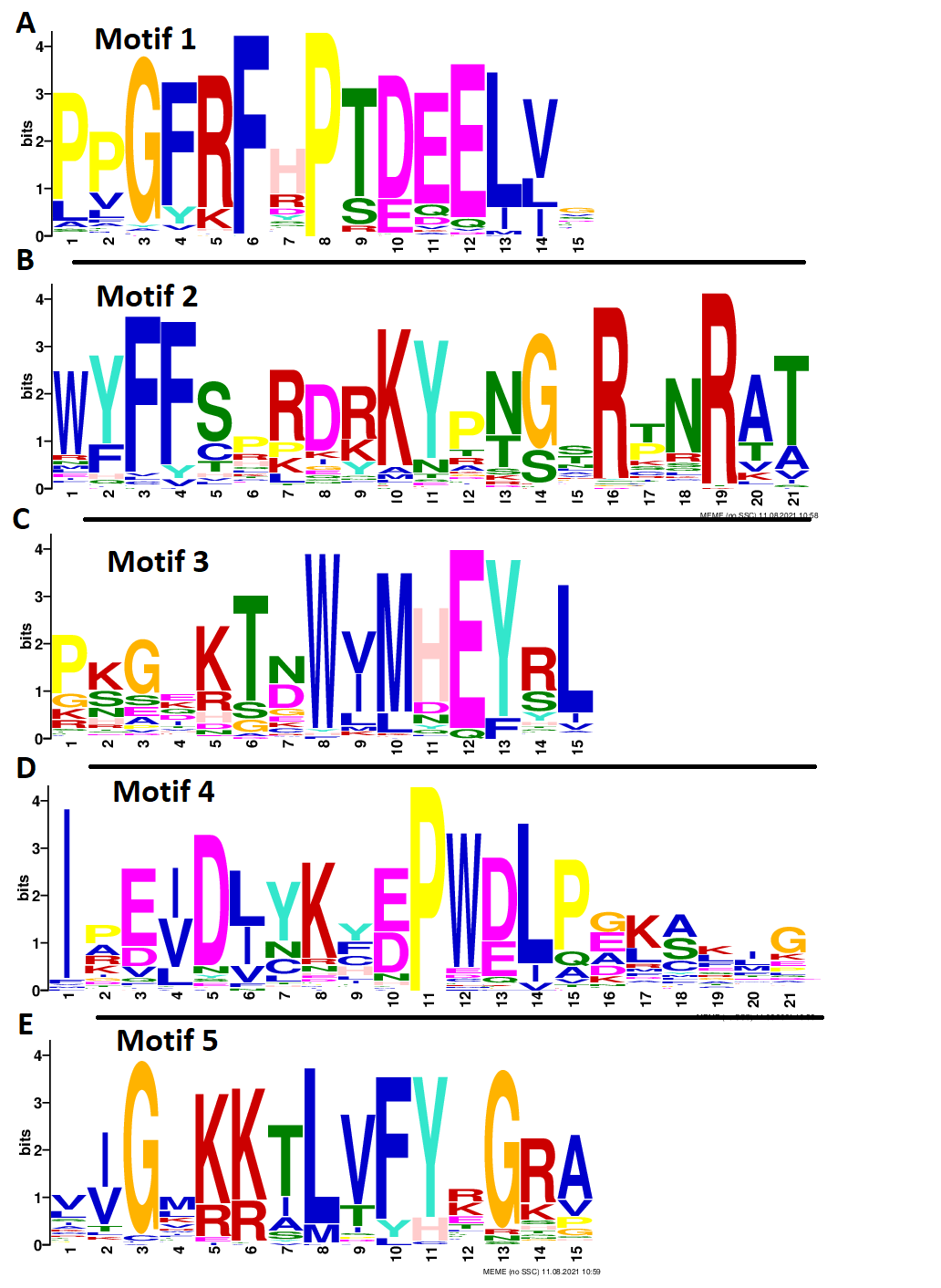

Supplement: Supplementary file 1 [file plants-10-02200-s001.zip › Supplementary Files/Supplementary Figure S2.tif]

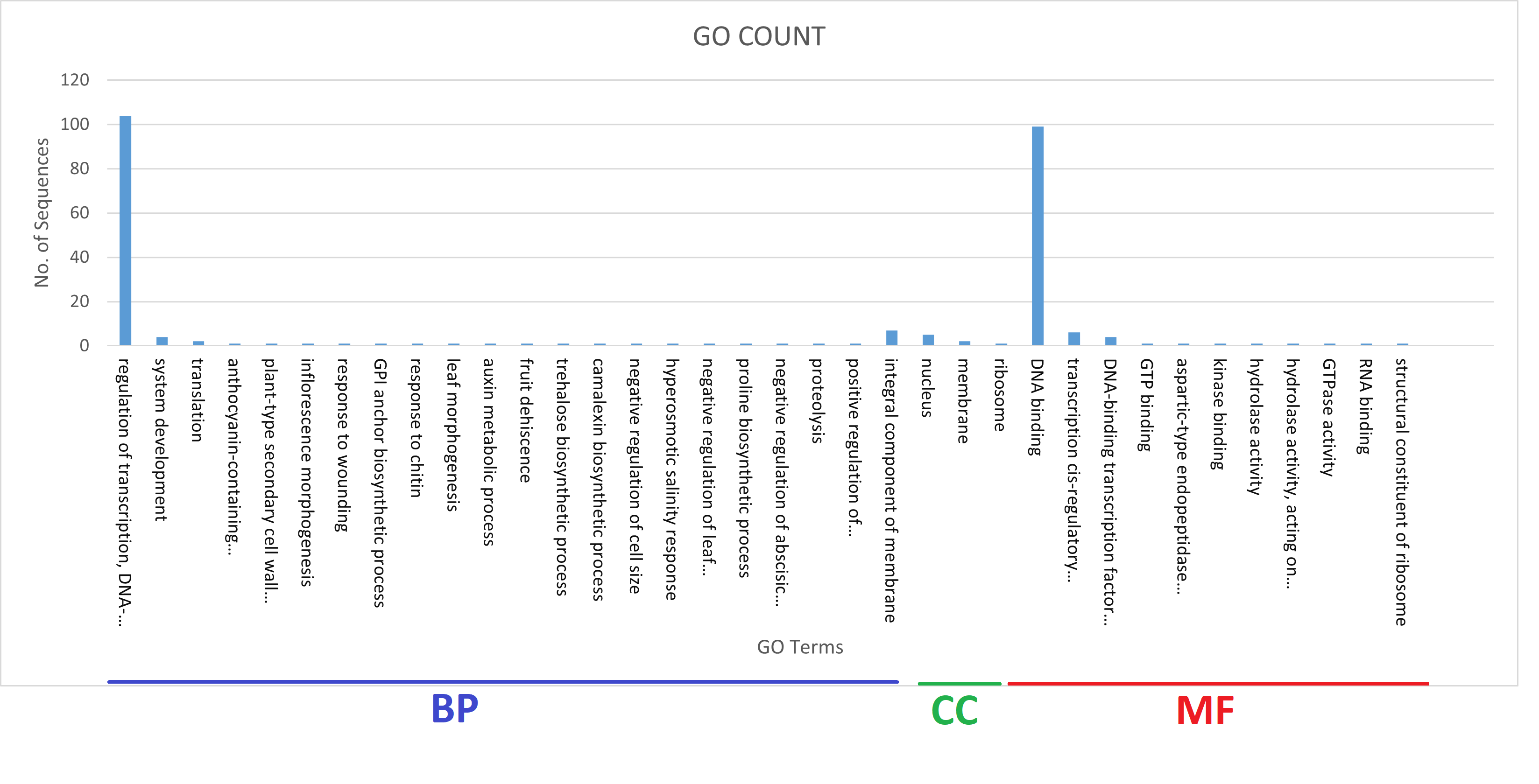

Supplement: Supplementary file 1 [file plants-10-02200-s001.zip › Supplementary Files/Supplementary Figure S3.tif]

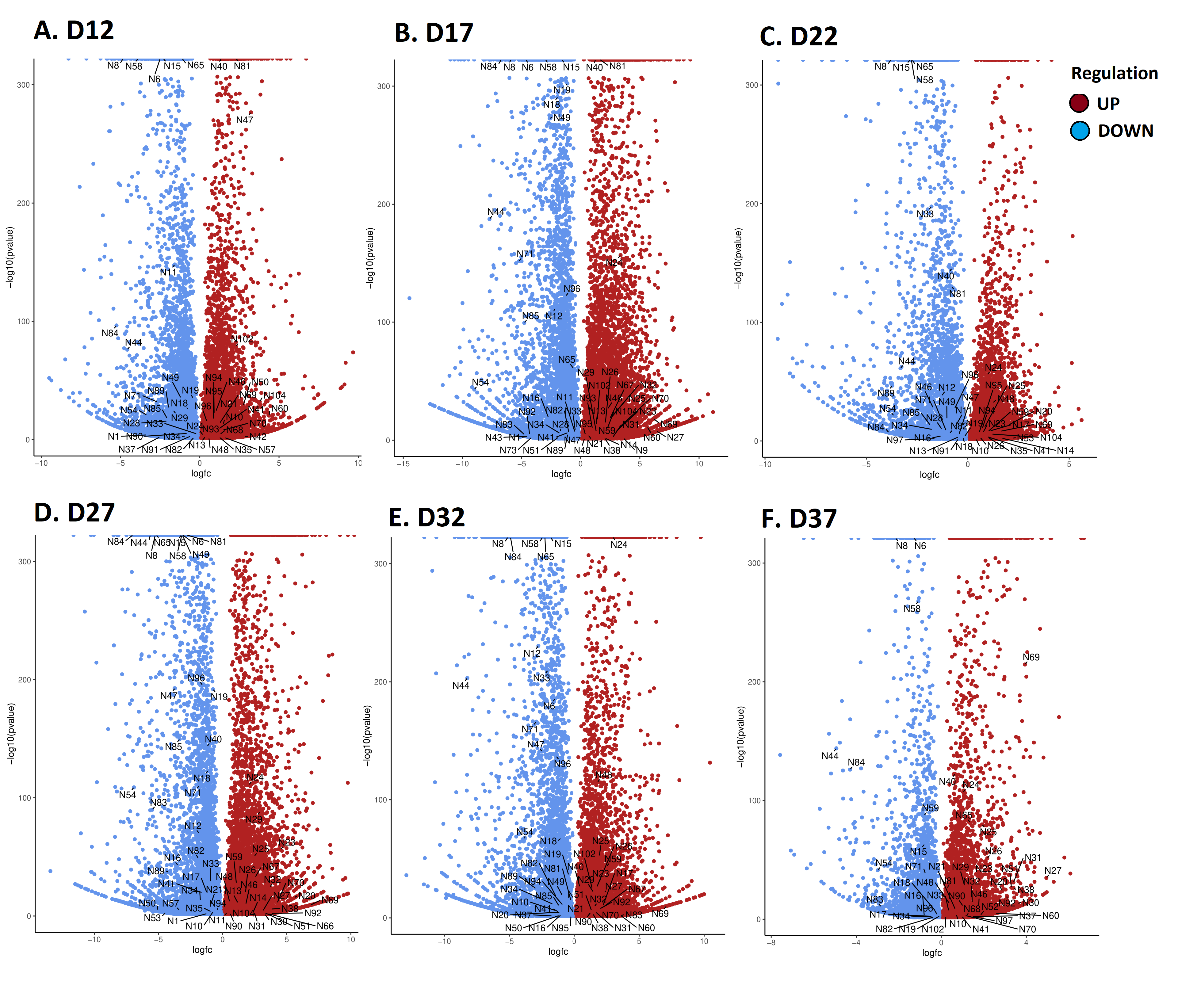

Supplement: Supplementary file 1 [file plants-10-02200-s001.zip › Supplementary Files/Supplementary Figure S4.tif]

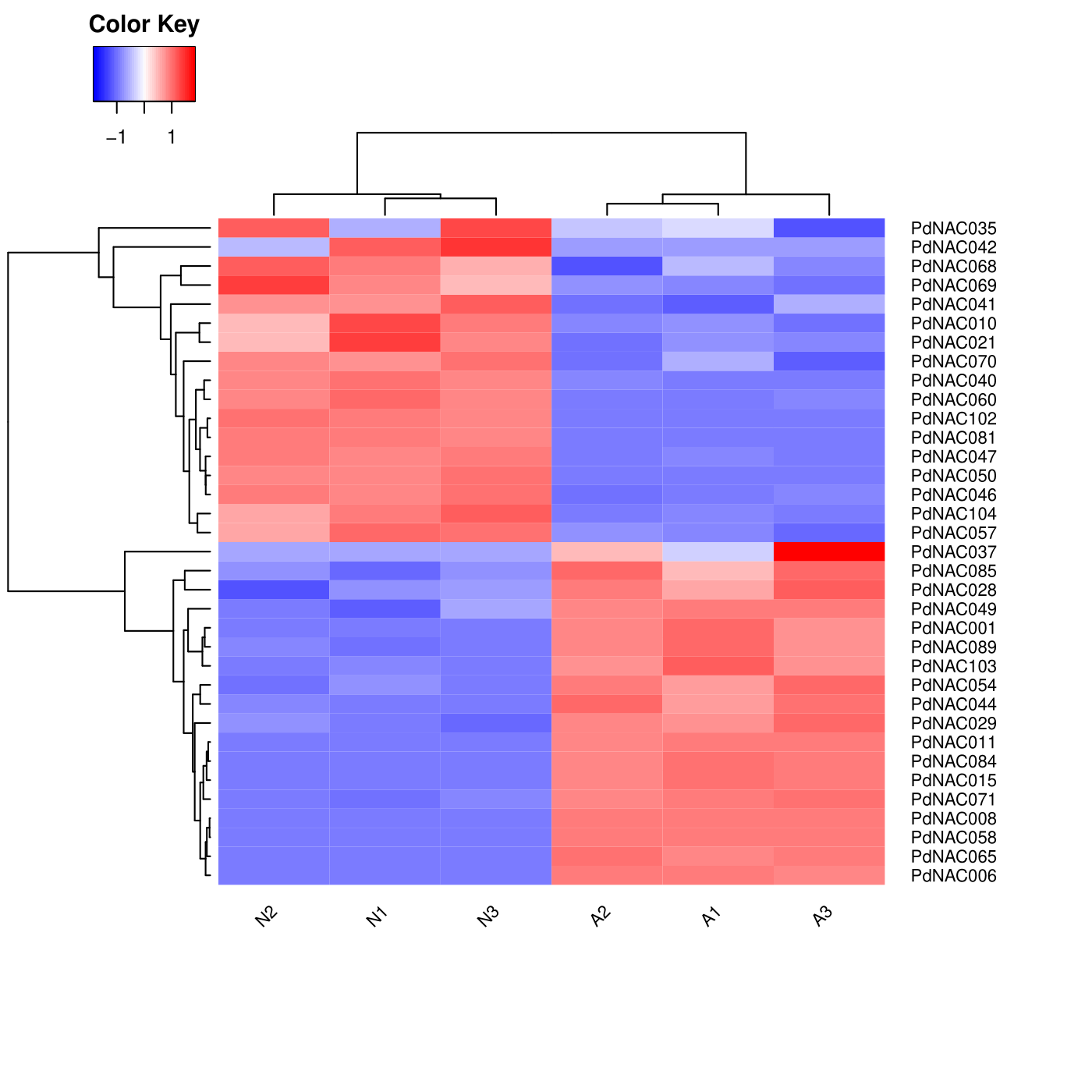

Supplement: Supplementary file 1 [file plants-10-02200-s001.zip › Supplementary Files/Supplementary Figure S5.tiff]

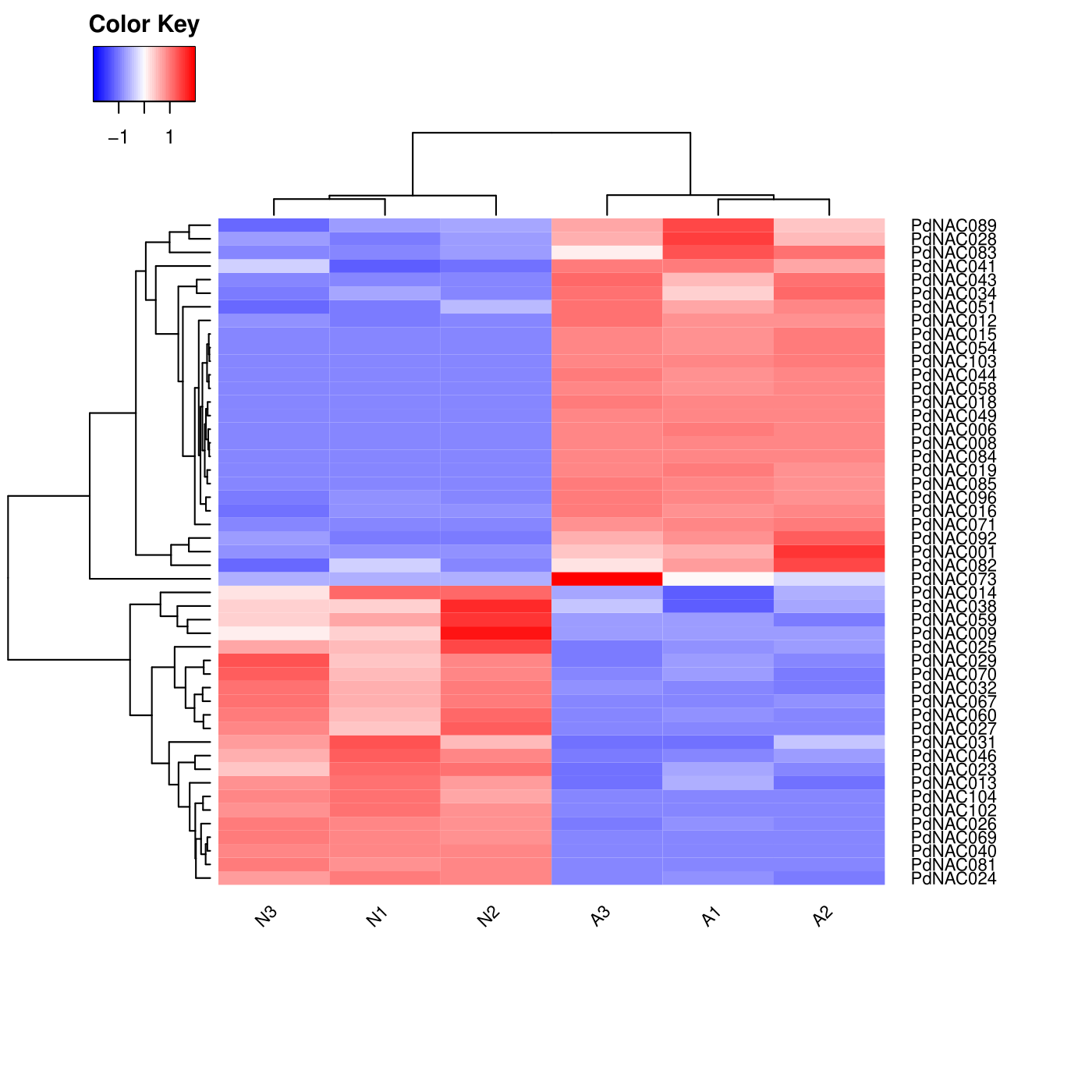

Supplement: Supplementary file 1 [file plants-10-02200-s001.zip › Supplementary Files/Supplementary Figure S6.tiff]

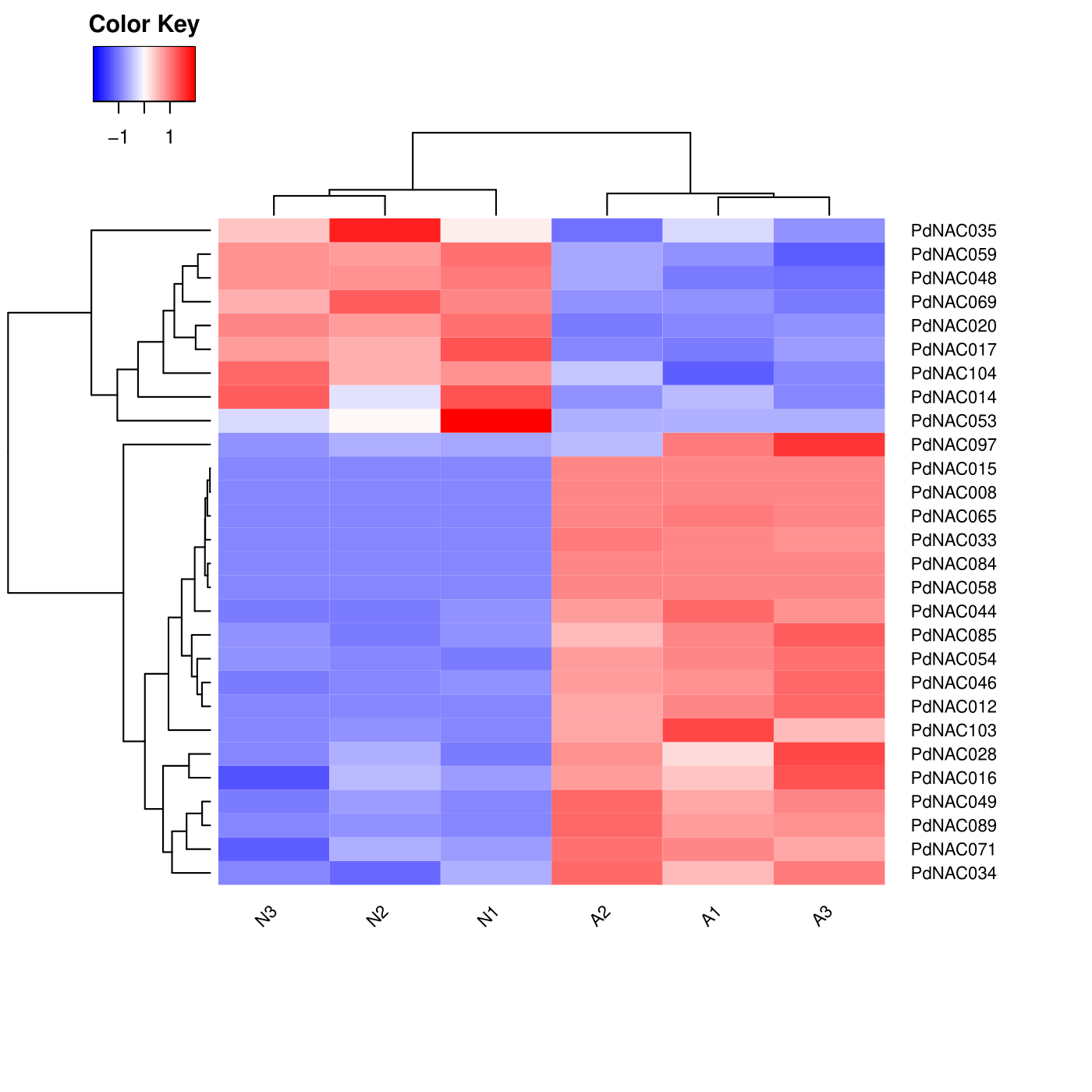

Supplement: Supplementary file 1 [file plants-10-02200-s001.zip › Supplementary Files/Supplementary Figure S7.tiff]

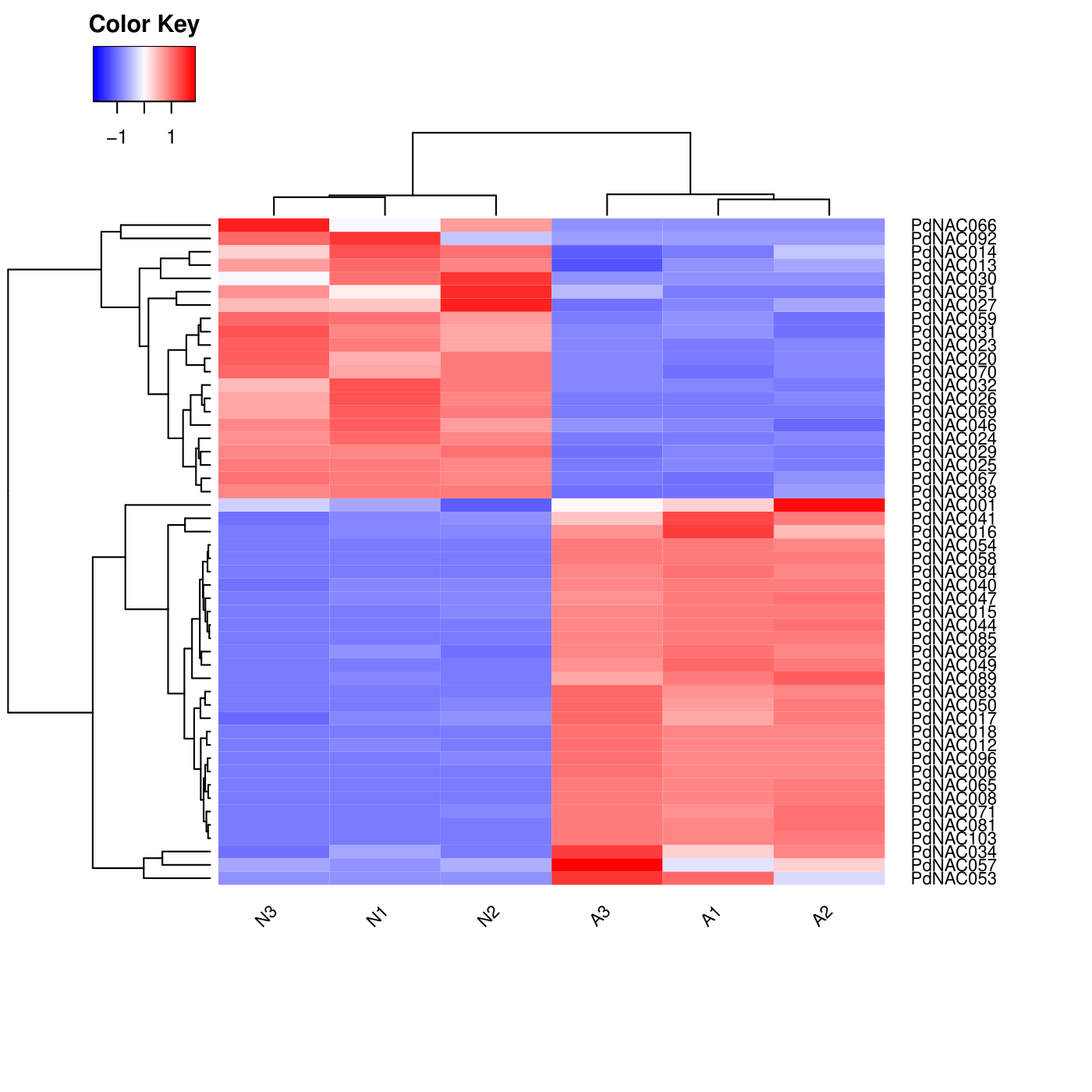

Supplement: Supplementary file 1 [file plants-10-02200-s001.zip › Supplementary Files/Supplementary Figure S8.tiff]

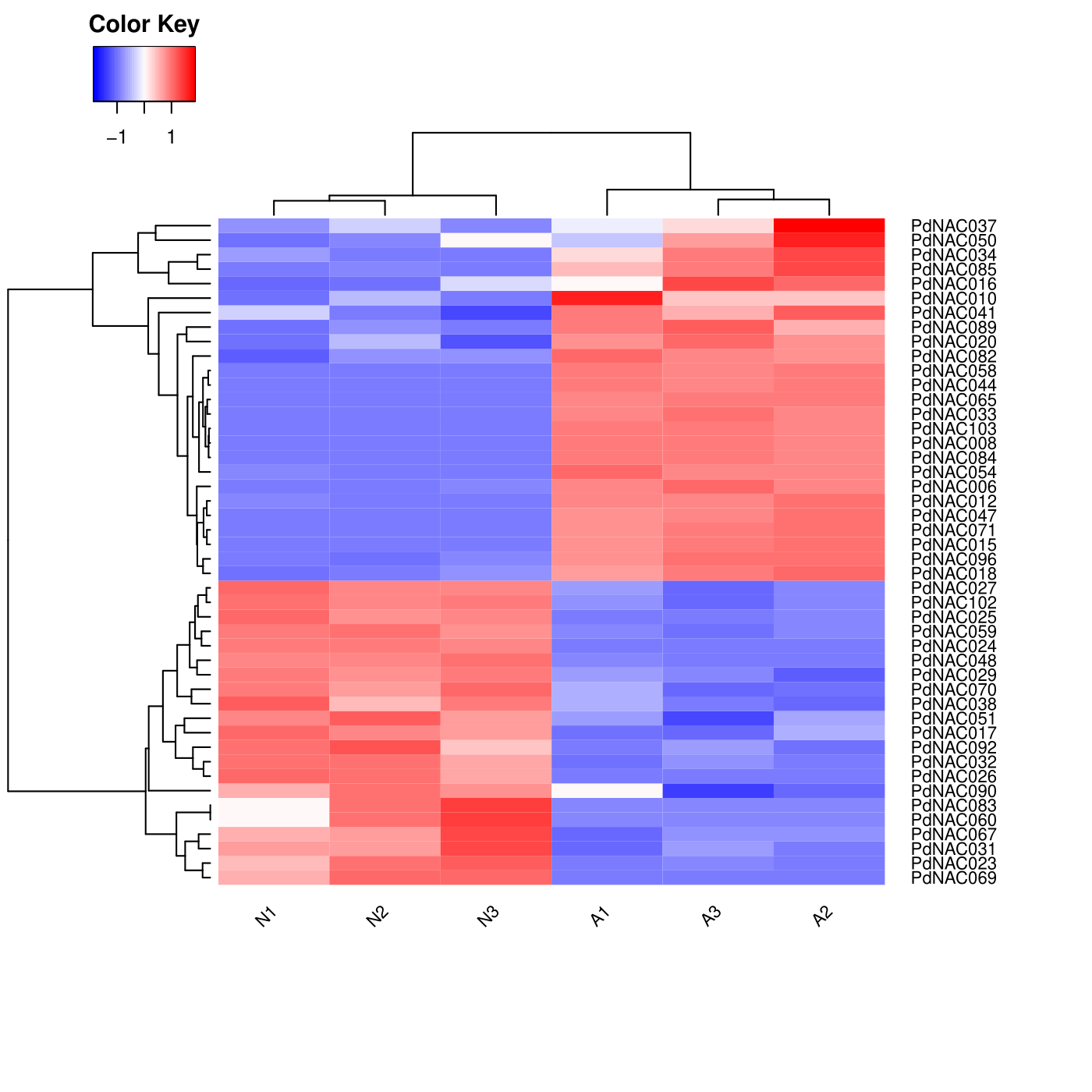

Supplement: Supplementary file 1 [file plants-10-02200-s001.zip › Supplementary Files/Supplementary Figure S9.tiff]
